# Huang-Lian-Jie-Du decoction alleviates cognitive impairment in periodontitis rats through restoring microbiota-gut-brain axis and inhibiting neuroinflammation via TLR4/NF-κB pathway

**DOI:** 10.1186/s13020-025-01235-6

**Published:** 2025-10-23

**Authors:** Mingqi Chen, Jie Li, Pan Ren, Sixiang Yang, Furong Zhong, Yue Zhu, Yiran Fan, Jinxin Chen, Manru Xu, Wenbin Wu

**Affiliations:** 1 Department of Geriatrics, Hospital of Chengdu University of Traditional Chinese Medicine, Chengdu, China; 2 Key Laboratory of Sichuan Province Ophthalmopathy Prevention & Cure and Visual Function Protection With TCM, Chengdu University of Traditional Chinese Medicine, Chengdu, China

**Keywords:** Cognitive impairment, Huang-Lian-Jie-Du decoction, Microbiota-Gut-Brain axis, Neuroinflammation, Periodontitis

## Abstract

**Background:**

Huang-Lian-Jie-Du decoction (HLJDD), a typical formulation for heat clearance and detoxification, shows therapeutic potential for oral diseases and cognitive impairment. Nevertheless, the mechanism by which HLJDD influences periodontitis-induced cognitive impairment via the microbiota-gut-brain axis remains unknown.

**Aim of the study:**

We investigated HLJDD’s neuroprotective effects in periodontitis rats, focusing on its modulation of the microbiota-gut-brain axis and underlying molecular mechanisms.

**Materials and methods:**

Chemical profiling of HLJDD was performed via UHPLC-Q-Exactive Orbitrap HRMS. Periodontitis was induced in SD rats using ligatures and *Porphyromonas gingivalis* for 2 weeks, followed by 8-week treatments with HLJDD (0.75/1.5/3 g/kg/day), doxycycline (10 mg/kg/day), or vehicle. Alveolar bone loss was assessed via micro-CT, while cognitive function was assessed via the Morris water maze (MWM). Hippocampal and colon pathology was analyzed via H&E, Nissl staining, and immunohistochemistry. The composition of gut microbiota was analyzed by 16S rDNA sequencing. The tight junction proteins in hippocampus and colon were examined by RT- qPCR and immunofluorescence (IF). Inflammatory cytokine levels in intestinal and hippocampus tissue and serum were quantified by ELISA. Network pharmacology predicted potential mechanisms, and Western blotting assessed TLR4/NF-κB pathway proteins.

**Results:**

HLJDD contained 94 bioactive compounds and significantly attenuated alveolar bone loss, improved cognitive function, and reduced neuronal damage and Aβ deposition. It restored gut microbiota homeostasis, enhanced intestinal and blood–brain barrier integrity, and suppressed neuroinflammation by modulating pro- and anti-inflammatory cytokines. Mechanistically, HLJDD inhibited TLR4/NF-κB signaling, suggesting its therapeutic potential in periodontitis-related cognitive impairment.

**Conclusion:**

HLJDD ameliorates cognitive impairment in periodontitis by modulating the microbiota-gut-brain axis, reducing neuroinflammation, and inhibiting TLR4/NF-κB activation. These findings support its potential as a novel therapeutic strategy for periodontitis-associated cognitive impairment.

**Graphical Abstract:**

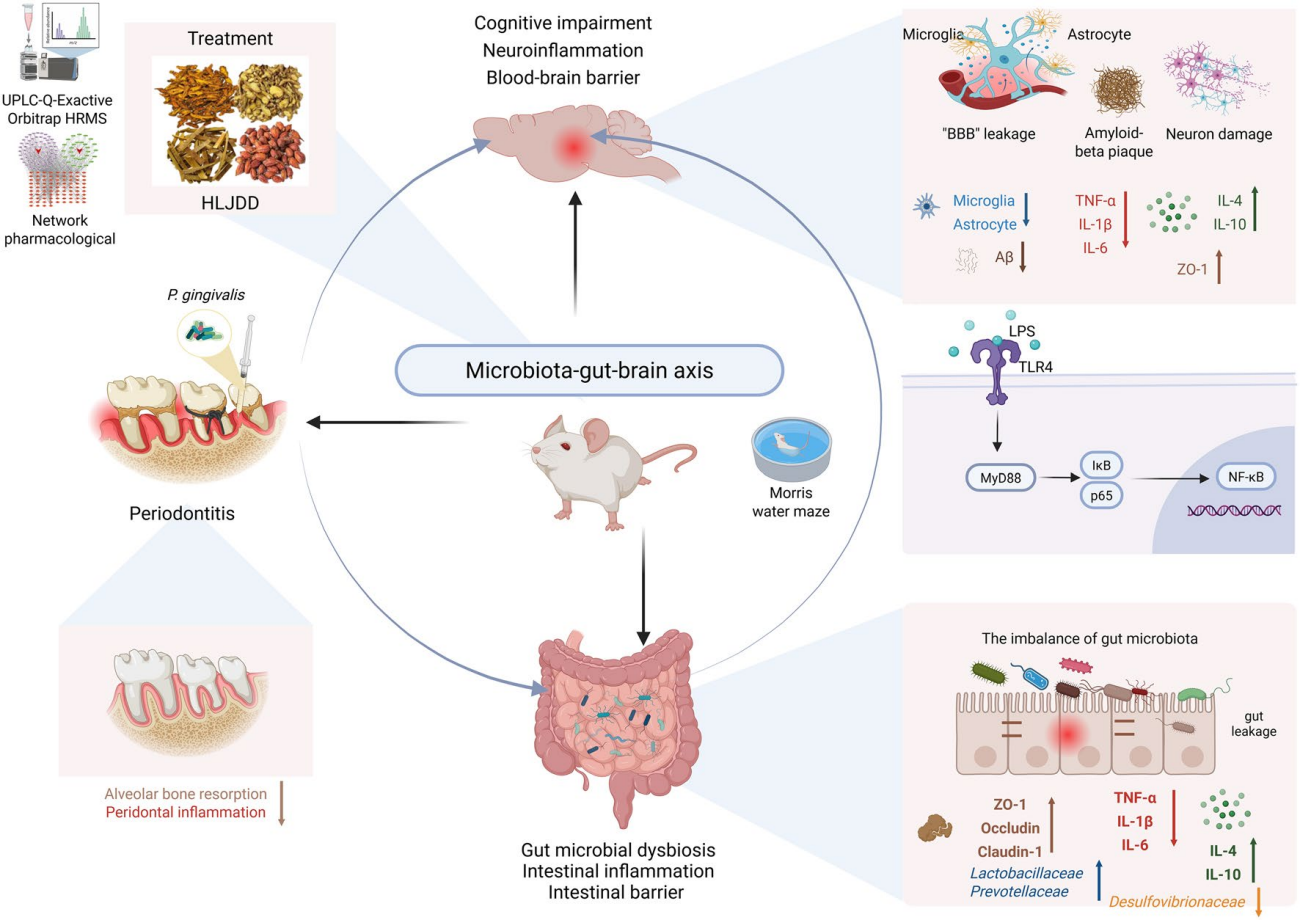

**Supplementary Information:**

The online version contains supplementary material available at 10.1186/s13020-025-01235-6.

## Introduction

Periodontitis (PD) is, a chronic inflammatory disease with substantial morbidity, characterized by the gradual destruction of periodontal tissues, including gingival inflammation and alveolar bone resorption, potentially resulting in tooth loss in advanced cases [1, 2]. Growing evidence suggests that periodontitis may serve as a risk factor for multiple systemic ailments, including respiratory, cardiovascular, and certain malignancies, as well as neurodegenerative diseases [3–6]. Recently, particular attention has focused on the probable connection between periodontitis and cognitive decline. Cheng-En Sung et al. demonstrated a significant correlation between periodontal status and cognitive impairment in U.S. adults through analysis of NHANES III data [7]. Similarly, Rong et al. reported patients with periodontitis had a higher risk of developing Alzheimer's disease (AD) [8]. These findings collectively highlight the significance of periodontitis prevention and treatment as potential strategies for mitigating cognitive decline.

The pathogenesis of periodontitis-related cognitive impairment is complex, involving glymphatic dysfunction, immune dysregulation, microbiota dysbiosis, systemic inflammation, and neuroinflammation [9, 10]. The results of recent studies have shown that there is a substantial correlation between microbial dysbiosis and both periodontitis and cognitive impairment [11, 12], with gut-brain axis disruption and neuroinflammation playing pivotal roles in this relationship [13]. Under pathological conditions, such as periodontitis and gingivitis, oral microbes can enter the gastrointestinal tract via saliva, potentially altering gut microbiota composition in terms of richness and diversity [14]. Furthermore, gut microbiota dysbiosis promotes the proliferation of an excessive number Gram-negative bacteria, which could disrupt intestinal barrier’s integrity and induce an increase of toxic substances [15, 16]. This allows these substances to enter systemic circulation, triggering inflammation and elevating serum proinflammatory cytokine levels [17]. Additionally, these inflammatory mediators can compromise blood–brain barrier (BBB) permeability and excessively activate microglia and astrocytes through inflammatory pathways [12]. Simultaneously, several research have revealed that periodontitis-induced cognitive dysfunction may be linked to the microbiota-gut-brain axis disruption, TLR4/NF-κB signaling activation, and neuroinflammatory responses [18]. Collectively, these findings suggest that periodontitis contributes to cognitive impairment and neuronal injury via microbiota-gut-brain axis dysregulation and neuroinflammation.

Huang-Lian-Jie-du decoction (HLJDD), originating from the Eastern Jin Dynasty’s “Handbook of Prescriptions for Emergency”, serves as a prominent formula for heat clearance and detoxification, comprised of Coptidis rhizoma, Scutellariae radix, Phellodendri chinensis cortex and Gardenia fructus. HLJDD treatment has demonstrated efficacy in addressing inflammatory, gastrointestinal, and neurodegeneration diseases, as evidenced by both clinical and animal experiments [19–21]. Experimental evidence suggests that HLJDD exerts neuroprotective effects through modulation of inflammatory pathways. Tian et al. reported that HLJDD treatment attenuated cognitive impairment in diabetic mice by suppressing neuroinflammation [22]. Furthermore, Yuan et al. demonstrated that HLJDD administration restored gut microbial homeostasis, ameliorated colonic tissue damage, and exerted anti-inflammatory effects in murine models of ulcerative colitis [23]. Additionally, Baicalin has been found to reduce IL-6 and TNF-α levels, ameliorated alveolar bone loss, and restored ileal permeability in aging-periodontitis mice by modulating the gastrointestinal microbiota and metabolites [24]. These studies indicate that HLJDD is effective against chronic inflammatory diseases by regulating gut microbiome or inhibiting inflammatory response. However, the potential neuroprotective effects of HLJDD on periodontitis-related cognitive impairment remain unexplored, potentially offering more significant clinical implications for cognitive impairment compared to targeted treatments.

We hypothesize that HLJDD alleviates cognitive impairment in PD rats through modulating the microbiota-gut-brain axis and inhibiting neuroinflammation. To test this, we established a ligature-adhered *Porphyromonas gingivalis* (*P. gingivalis)* application periodontitis model in SD rats. The effects of HLJDD were assessed on several parameters including: (1) alveolar bone resorption, (2) cognitive function, (3) neuron, (4) gut microbiota composition, (5) the integrity of both intestinal and blood–brain barriers, and (6) intestinal and neuroinflammation. Additionally, we aimed to uncover the potential mechanisms underlying these effects.

## Materials and methods

### Preparation and UHPLC-Q-Exactive Orbitrap HRMS analysis of HLJDD

HLJDD consisted of the following ingredients, which acquired from Sichuan NEAUTUS Traditional Chinese Medicine Co., Ltd (Chengdu, China): Coptidis rhizome (Huanglian), Scutellariae radix (Huangqin), Phellodendri chinensis cortex (Huangbai), Gardenia fructus (Zhizi) (Table 1). All the herbs were immersed in water at a ratio of 1:10 (herbs to water) and allowed to soak for 30 min, then refluxed and extracted three times, with each extraction boiling for 30 min. The extract was subsequently concentrated to a therapeutic equivalent of 0.3 g/ml, portioned, and stored at -20℃ until use.

**Table 1:** Composition of HLJDD

Chinese name	Pharmaceutical name	Botanical name	Batch number	Weight (g)
Huanglian	Coptidis rhizoma	*Coptis chinensis* Franch	2,306,085	9
Huangqin	Scutellariae radix	*Scutellaria baicalensis* Georgi	2,305,034	6
Huangbai	Phellodendri chinensis cortex	*Phellodendron Chinense* Schneid	2,308,321	6
Zhizi	Gardenia fructus	*Gardenia jasminoides* Ellis	2,310,067	9

The chemical profiling of HLJDD extract was conducted using ultra-high performance liquid chromatography coupled with quadrupole-Orbitrap high-resolution mass spectrometry (UHPLC-Q-Exactive Orbitrap HRMS). The analysis was performed on an ACQUITY UPLC I-Class Plus system (Waters Corporation, USA) equipped with a Q Exactive mass spectrometer featuring a heated electrospray ionization (HESI) source (Thermo Fisher Scientific, USA). Chromatographic separation was achieved using an ACQUITY UPLC HSS T3 column (2.1 × 100 mm, 1.8 μm) with a binary mobile phase system consisting of 0.1% aqueous formic acid (A) and acetonitrile (B). The gradient elution program was optimized as follows: 0–3 min (0% B), 3–18.5 min (0–20% B), 18.5–20 min (20–35% B), 20–26 min (35–40% B), 26–35 min (40–95% B), 35–38 min (95% B), and 38.1–40 min (0% B), with a constant flow rate of 0.35 mL/min at 45 °C column temperature and 2 μL injection volume. Mass spectrometric detection was performed in both positive and negative ionization modes with the following HESI parameters: spray voltages of + 3.8 kV (positive) and − 3.2 kV (negative), sheath gas flow of 35 arb, auxiliary gas flow of 8 arb, heater temperature at 350 °C, and ion transfer tube temperature at 320 °C. Full-scan MS data were acquired over m/z 100–1500 range at 60,000 resolution, complemented by data-dependent MS^2^ scans at 15,000 resolution. All acquired data were processed and analyzed using XCMS software (version 4.5.1) for comprehensive compound identification.

### Animals

Male Sprague–Dawley (SD) rats that were eight weeks old and obtained from Chengdu Dossy Experimental Animals Co., Ltd. (Chengdu, China) were used in this investigation. The rats weighed between 200 and 250 g on average and were specific pathogen-free (SPF). They had free access to food and water while housing in SPF conditions at the College of Pharmacy, Chengdu University of Traditional Chinese Medicine (Chengdu, China). The Medical Ethics Committee of the Hospital of Chengdu University of Traditional Chinese Medicine authorized all experimental procedures, which followed the “3R” principles (Approval No. 2024DL-014; Chengdu, China).

### Periodontitis model and intervention

**Fig. 1:**
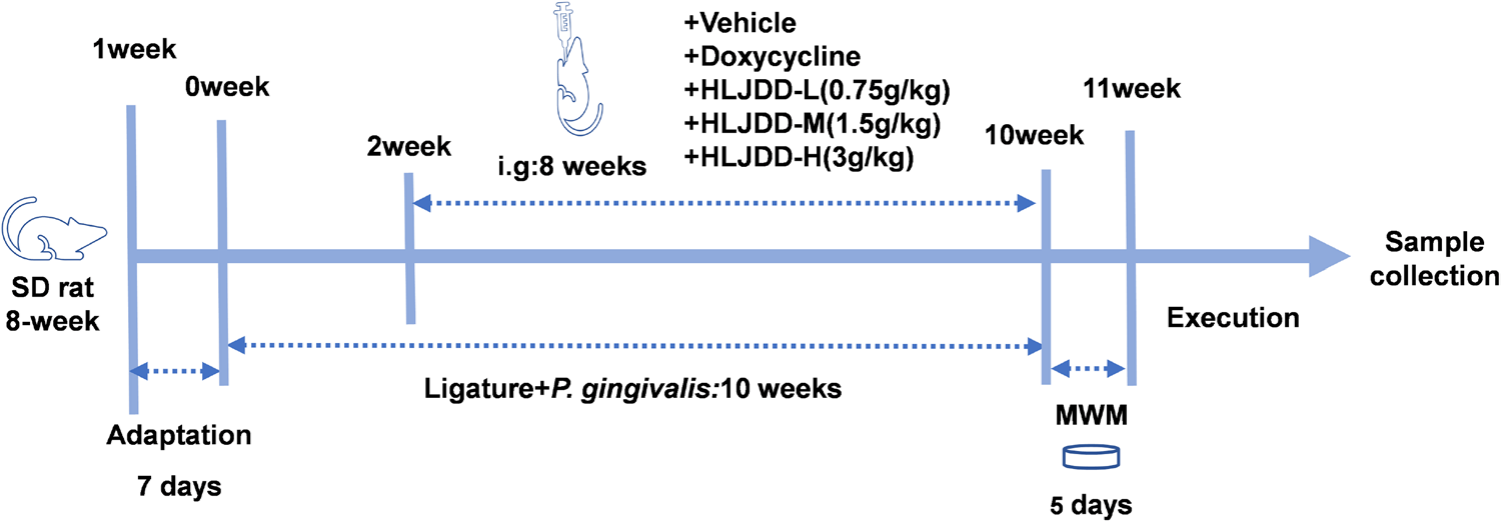
Flowchart of experimental design

Following one week of acclimatization, all SD rats were randomly assigned to six groups (n = 8 per group): (1) the control (CTL) group; (2) the periodontitis model (PD) group, which received vehicle treatment; (3) the Doxy group, treated with 10 mg/kg doxycycline hyclate (412S031, Solarbio, Beijing, China); (4) the HLJDD-L group, administered 0.75 g/kg HLJDD; (5) the HLJDD-M group, given 1.5 g/kg HLJDD; and (6) the HLJDD-H group, receiving 3 g/kg HLJDD. The optimal high dose of 3 g/kg was determined through preliminary experiments. A medium dose of 0.3 g/kg is equivalent to 6.25 times the standard human clinical dose (adjusted for a 60 kg adult). Low and high doses were set at 1.5 g/kg (½ ×) and 6 g/kg (2 ×), respectively. Subsequent experimental results indicated that the 3 g/kg dose yielded the most significant improvement in MWM performance, accompanied by reduced serum inflammatory factors and decreased *P. gingivalis* copies in the hippocampus. The experimental timeline and design are presented in Fig. 1.

All the experimental rats, except for the CTL group, were given general anesthesia through an injection of 2% pentobarbital sodium (45 mg/kg; Merck, cat. no. 230816, NJ, USA). Using a mouth opener, we exposed each rat's oral cavity to visualize the bilateral maxillary molars after achieving adequate anesthesia. A 4–0 silk suture was threaded through the gaps next to the second maxillary molar and tied with a surgical knot on the outer side, and *P. gingivalis* (ATCC 33277) was applied just below the gum line. We assessed the integrity of the ligature three times a week, re-ligating it immediately when necessary. In addition, *P. gingivalis* was applied subgingivally once every other day. Following a two-week model establishment period, all animal groups received daily oral gavage administrations (10 ml/kg) for 8 consecutive weeks. The modeling stimulation were maintained throughout the entire intervention period, resulting in a total experimental duration of 10 weeks. Upon study completion, rats were euthanized via pentobarbital, and then their mandibular, hippocampal, and colon tissues, along with intestinal contents and blood, were collected for further analysis.

### Micro-Computed Tomography (micro-CT) Imaging

Mandibular specimens were surgically excised from euthanized rats and examined via micro-computed tomography (micro-CT) using a Quantum GX2 scanner (PerkinElmer, USA). The scanning parameters includedt 90 kV voltage, 80 μA current, 4-min acquisition time, an 18 mm field of view, and 36.0 μm isotropic voxel size. Three-dimensional (3D) reconstructions were generated using Auto Viewer Analyze 12.0 software. To quantify alveolar bone loss, we measured the linear distance between the cemento-enamel junction (CEJ) and alveolar bone crest (ABC) at the maxillary second molars.

### Behavioral Analysis -Morris Water Maze (MWM) Test

All SD rats were given the MWM test to assess their spatial learning and memory after finishing the gavage treatment. A circular pool (120 cm in diameter and 55 cm in depth) containing a submerged platform (1 cm below water surface; Taimeng Technology, Chengdu, China). The examination comprised two phases: (1) a 4-day acquisition phase where rats learned platform location through four daily trials (one from each quadrant), with manual guidance to the platform after 60 s if needed, followed by a 10-s platform stay; and (2) a probe experiment in which the platform was withdrawn and rats had 60 s to demonstrate spatial memory. Using the MWT-100 video tracking system (Taimeng Technology), the performance was captured and analyzed.

### Hematoxylin & eosin (H&E) staining

Using a microtome, tissue samples from the colon and hippocampal regions were sliced into 5 μm slices after being preserved in 4% paraformaldehyde for 24 h, subsequently dehydrated, and embedded in paraffin wax. Haematoxylin and eosin were applied to stain the finished slices after they had been dewaxed with xylene and run through an aqueous ethanol series. Ultimately, the slices were analyzed and recorded with the Slide Scanning System SQS-12P (Shenzhen Shengqiang Technology Co., Ltd., Shenzhen, China).

### Nissl staining

Hippocampal sections were prepared following the H&E staining protocol. The sections underwent staining with 0.1% toluidine blue solution at a temperature of 50 °C, followed by an incubation period at 56 °C for 20 min. After air-drying, the sections were coverslipped using a neutral balsam mounting medium. Nissl bodies located in the hippocampus (CA1, CA3, and DG regions) were visualized and imaged using an SQS-12P slide scanning system.

### Immunohistochemistry (IHC)

Hippocampal slices were collected as described in the H&E staining steps above. After dewaxing and antigen retrieval, sections were washed in phosphate-buffered saline (PBS) and incubated overnight at 4 °C with a primary antibody taigeting APP/β-amyloid (25,524–1-AP, 1:200 dilution; Proteintech). Following PBS washes, sections were incubated with secondary antibody for 20 min at room temperature. After DAB development and hematoxylin counterstaining, sections were dehydrated through a serie of ethanol solutions, cleared in xylene, and mounted with resin. Immunostained sections were imaged using an SQS-12P slide scanning system, and quantitative analysis was performed using ImageJ software (v1.54f; NIH, USA).

### Immunofluorescence (IF) assay

Hippocampal and colon tissue silces were dewaxed and rehydrated with citrate–phosphate buffered saline (CPBS; PH6.0, ZSGB-BIO). Following a 30-min incubation with 3% bovine serum albumin (BSA; Servicebio), slices were treated overnight at 4 °C with primary antibodies (Table 2). On the next day, slices were treated for 1 h at ambient temperature with the secondary antibodies: Cy3-conjugated goat anti-mouse IgG (1:200, Servicebio) and FITC-conjugated goat anti-rabbit IgG (1:300, Servicebio). Sections were mounted with anti-fade medium (Servicebio) and imaged using an OLYMPUS OlyVIA 4.1 system. Quantitative analysis was applied by ImageJ software (v1.54f; NIH, USA).

**Table 2:** Primary antibodies

Antibody	Dilution	Application		Source
Iba1	1:200	IF	10,904–1-AP	Proteintech
GFAP	1:200	IF	60,190–1-lg	Proteintech
ZO-1	1:100	IF	AF5145	Affinity Biosciences
Occludin	1:100	IF	DF7504	Affinity Biosciences
Claudin-1	1:100	IF	AF0127	Affinity Biosciences
TLR4	1:2000	WB	AF7017	Affinity Biosciences
MyD88	1:2000	WB	AF5195	Affinity Biosciences
Phospho-NF-κB p65	1:2000	WB	AF2006	Affinity Biosciences
NF-κB p65	1:2000	WB	AF5006	Affinity Biosciences
Phospho-IKB-α	1:2000	WB	AF2002	Affinity Biosciences
IKB-α	1:2000	WB	AF5002	Affinity, China
β-actin	1:5000	WB	AF7018	Affinity, China

### Enzyme-linked immunosorbent assay (ELISA)

The quantities of TNF–α, IL–1β, IL–4, IL–6, and IL–10 was measured in the hippocampal and colon tissues, as well as in serum samples, utilizing a rat ELISA kit provided by Jiangsu Jingmei Biological Technology Co., Ltd. (Jiangsu, China) in compliance with the guideline.

### Western blot (WB)

Using specific primary antibodies (Table 2), Western blot analysis was carried to measure the amount of the target protein present in hippocampal tissues. Following the instructions, a BCA protein test kit (Cat# 23,227; Thermo Fisher Scientific, USA) was applied to measure the total protein quantity. The bands detected utilizing a JY-Clear ECL chemiluminescence imaging system (Beijing JUNYI Electrophoresis Co., Ltd).

### Real-time quantitative polymerase chain reaction (RT-qPCR)

Using an RNAiso Plus Fast Tissue Kit (Takara Biomedical Technology Co. Ltd., Beijing, China), RNA was extracted from rat colonic tissue in accordance with the instructions. The RT Easy™ II Kit (containing gDNase) (FOREGENE, Chengdu, China) was then used to accomplish cDNA synthesis. Table 3 provides specifics on the primer pairings that were utilized.

**Table 3:** Primers sequences

Name	Forward Primer	Reverse Primer
ZO-1	5’-ATGACCGAGTCGCAATGGTT-3’	5’-AGCTGCTGAACAGCAAAAGC-3’
Occludin	5’-AGGTTACGGTTACGGCTA-3’	5’-ACCAAGGAAGCGATGAAG-3’
Claudin-1	5’-GGATCGGCTCTATCGACA-3’	5’-TCGTAGATGGCCTGAGCA-3’
β-actin-	5’-CCCGCGAGTACAACCTTCTT-3’	5’-CGCAGCGATATCGTCATCCA-3’

### 16S rRNA sequencing and data analysis

Colon content samples were processed to extract genomic DNA utilizing the TGuide S96 Magnetic Stool DNA Kit (Tiangen Biotech, Beijing, China) in accordance with the instructions. Primers 338F (5'-ACTCCTACGGGAGGCAGCA-3') and 806R (5'-GGACTACHVGGGTWTCTAAT-3') were utilized in order to amplify the V3–V4 region of the 16S rRNA genes found in bacteria. This was then followed by sequencing on an Illumina NovaSeq 6000 platform (Biomarker Technologies, Beijing). Raw sequencing data were processed as follows: (1) quality filtering using Trimmomatic (v0.33), (2) primer removal with Cutadapt (v1.9.1), and (3) analysis in QIIME2 (v2020.6) to generate high-quality reads. Operational taxonomic units (OTUs) were classified against the SILVA database (v138). Alpha diversity analysis, utilizing Chao1 index and Shannon indices, was performed with the OTU table in RStudio (v4.4.1) and shown as box plots. Principal coordinate analysis (PCoA) was applied to illustrate the results of a beta diversity analysis with Bray–Curtis dissimilarity. Additionally, to determine which taxa were differently prevalent across groups, the Linear Discriminant Analysis effect size (LEfSe) method was employed.

### Network pharmacological analysis

The chemical components of HLJDD were assessed via UHPLC-Q-Exactive Orbitrap HRMS, with compound structures sourced from PubChem. Potential targets were forecasted using SwissTargetPrediction, retaining entries with probability > 0. Common targets among HLJDD, periodontitis, and cognitive impairment were identified from GeneCards, OMIM, and TTD, followed by Venn diagram visualization. A compound–target–disease network was built in Cytoscape (v3.7.1/3.10.3) to depict therapeutic associations. Overlapping targets were used to establish a PPI network via STRING (v12.0). In Cytoscape, core targets were identified using the CentiScape 2.2 plugin by selecting nodes with values above the median in betweenness centrality, closeness centrality, and degree, followed by sorting based on degree. GO and KEGG enrichment analyses were performed in DAVID (v6.8), with top results displayed graphically.

### Statistical analysis

The data processing in this study was conducted utilizing two analytical software packages: GraphPad Prism 8.0 and RStudio 4.4.1. Quantitative results are expressed as arithmetic mean values with their corresponding standard errors (mean ± SEM). A one-way analysis of variance (ANOVA) was used for comparisons between different experimental groups, with statistical significance defined at the threshold of p-values less than 0.05.

## Results

### The chemical composition of the HLJDD extract

**Fig. 2:**
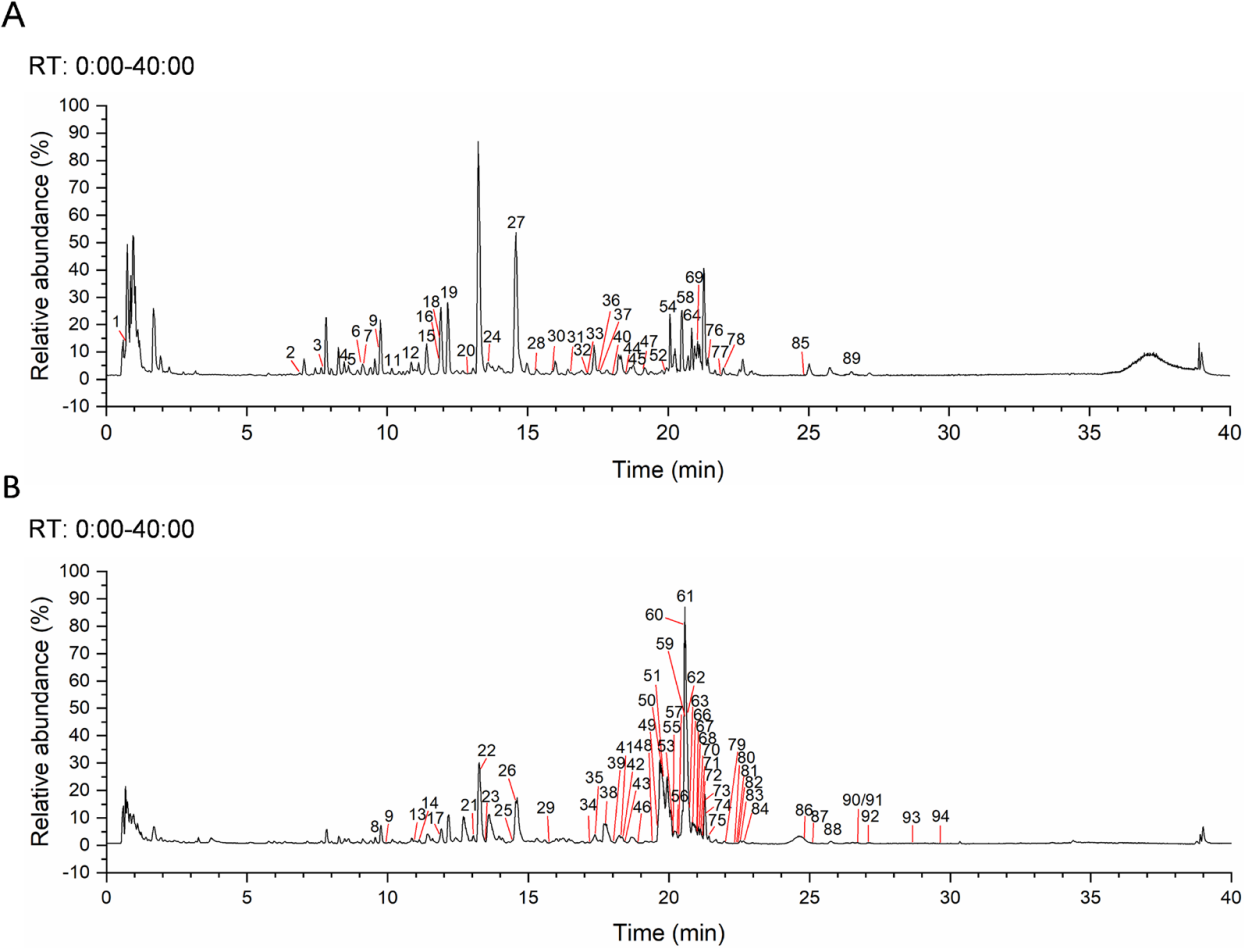
The ion chromatograms of HLJDD at (**A**) negative mode and (**B**) positive mode by UHPLC-Q-Exactive Orbitrap HRMS

The UHPLC-Q-Exactive Orbitrap HRMS was utilized to perform an analysis on the chemical components of HLJDD. The analysis was conducted in both positive and negative ionization modes, and the total run time involved was 40 min. The chemical composition of the HLJDD extract was identified based on chromatographic retention times and fragment ion peaks. By cross-referencing the results with online databases and relevant literature, we identified 94 major chemical components in the extract of HLJDD. Figure 2 and Table S1 display the detailed chemical compositions.

### HLJDD inhibits alveolar bone resorption in periodontitis rats

**Fig. 3:**
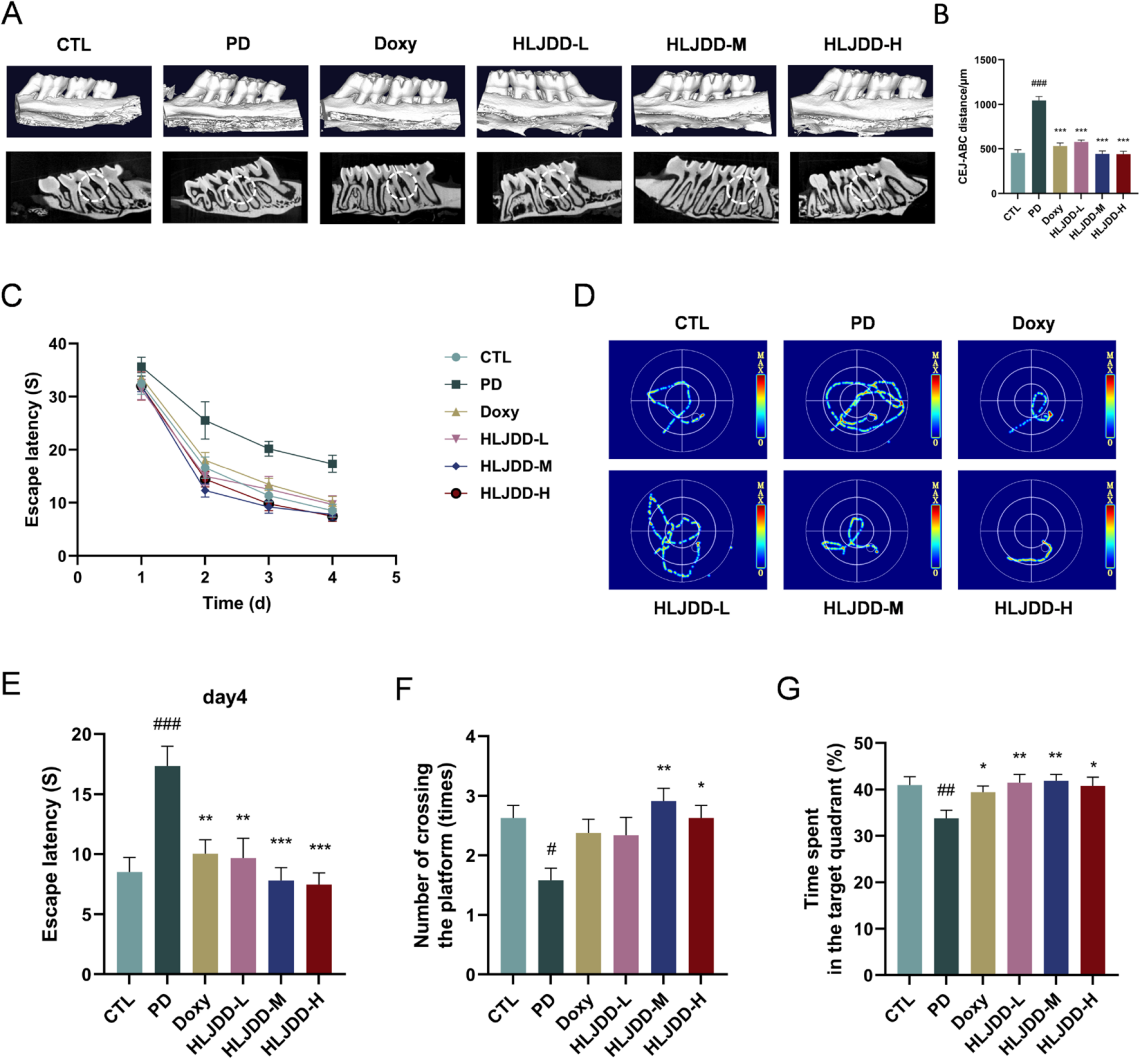
HLJDD treatment inhibits alveolar bone resorption and improves learning and memory ability in PD rats. **A** Micro-CT reconstructed 3D images of alveolar bone. (n = 3) (**B**) CEJ-ABC distance. (n = 3) (**C**) Escape latency. (n = 6) (**D**) Trajectory of swimming of day4. (n = 6) (**E**) Escape latency of day4. (n = 6) (**F**) The numbers of crossing the platform (times). (n = 6) (**G**) Time spent in the target quadrant (%). (n = 6) Data were expressed as the mean ± SEM. ^#^*P* < 0.05, ^##^*P* < 0.01, ^###^*P* < 0.001, vs. CTL group; ^*^P < 0.05, ^**^P < 0.01, ^***^P < 0.001, vs. PD group

Micro-CT analysis of the second upper molar after 8 weeks of treatment confirmed the therapeutic effect of HLJDD on alveolar bone loss in the furcation region. Compared to the CTL, Doxy, and HLJDD-L/M/H groups, the PD group exhibited significantly greater alveolar bone loss (Fig. 3A). Furthermore, the PD group had a significantly larger distance between the cemento-enamel junction (CEJ) and the alveolar bone (AB) apex than the CTL group (Fig. 3B). HLJDD treatment effectively attenuated alveolar bone loss and significantly restored alveolar ridge height in PD rats.

### HLJDD improves learning and memory capabilities in periodontitis rats

To examine how HLJDD protected the memory and cognitive function in PD rats, the MWM test was used. During the navigation test, all groups showed progressive reductions in escape latency across the four training days (Fig. 3C), with representative swimming trajectories illustrated in Fig. 3D. The PD group's escape latency was considerably greater than that of the CTL group on day 4. This impairment was significantly ameliorated in both Doxy and HLJDD-L/M/H groups (Fig. 3E). In the spatial probe test, PD rats exhibited substantially fewer platform crossings and reduced time spent in the target quadrant (%) compared to CTL rats. In comparison to the PD group, rats treated with HLJDD exhibited an extend duration at the initial platform location. However, HLJDD treatment increased the duration spent at the original platform location; the HLJDD-M and HLJDD-H groups showed significant effects (Fig. 3F-G). The findings presented here show that treating PD rats with HLJDD improves their memory and learning capacities.

### HLJDD decreases neuronal injury and APP/Beta amyloid accumulation in periodontitis rats

**Fig. 4:**
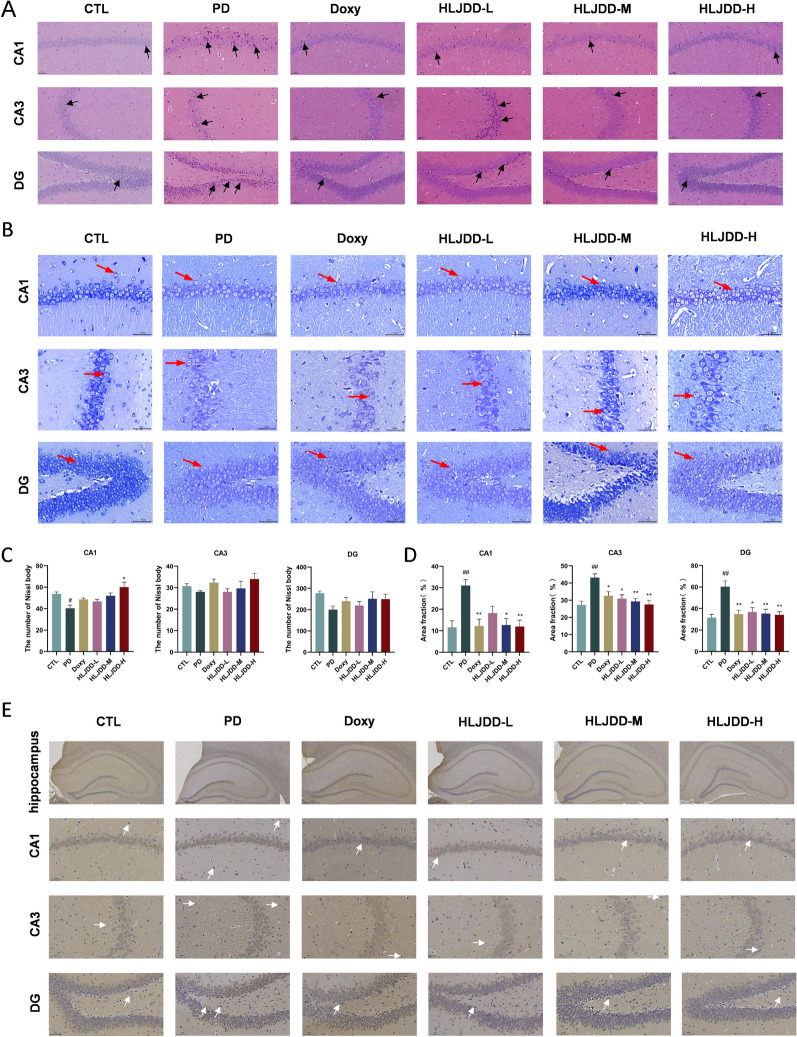
HLJDD treatment relieves neuronal injury and reduces Aβ accumulation in PD rats. **A** Representative images of H&E in the hippocampus. Black arrows indicate pyknotic and hyperchromatic nucleus. Scale bar = 50 µm. (n = 3) (**B**) Representative images of Nissl staining in the hippocampus. Red arrows indicate positive neurons). Scale bar = 50 µm. (n = 3) (**C**) The number of Nissl body in the CA1, CA3, and DG of the hippocampus. (n = 3) (**D**) Quantification of Aβ immunohistochemistry in the CA1, CA3, and DG of the hippocampus. White arrows indicate APP/Beta-amyloid deposits). (n = 3) (**E**) Representative images of Aβ immunohistochemistry in the hippocampus. Scale bar = 50 µm. (n = 3) Data were expressed as the mean ± SEM. ^#^*P* < 0.05, ^##^*P* < 0.01, ^###^*P* < 0.001, vs. CTL group; ^*^P < 0.05, ^**^P < 0.01, ^***^P < 0.001, vs. PD group

To evaluate the protective impact of HLJDD on neurons, we used H&E and Nissl staining to look more closely at the degenerative alterations in the hippocampus. H&E staining revealed abnormal neuronal morphology, including shrunken cells with pyknotic nuclei and neuronal loss, which were attenuated by HLJDD treatment (Fig. 4A). In comparison to the CTL group, Nissl staining revealed a notable decline in neuronal density in the PD rats' dentate gyrus (DG), CA1, and CA3 hippocampus regions. Neuronal survival in these hippocampus subregions was markedly protected by HLJDD treatment (Fig. 4B-E). In addition, IHC analysis showed elevated Aβ deposition in PD rats relative to CTL rats. HLJDD treatment significantly reduced Aβ accumulation in the CA1, CA3, and DG regions (Fig. 4F-I). Collectively, these findings demonstrate that HLJDD treatment effectively mitigates neuronal damage and reduces Aβ deposition in PD rats.

### HLJDD regulates gut microbiota in periodontitis rats

**Fig. 5:**
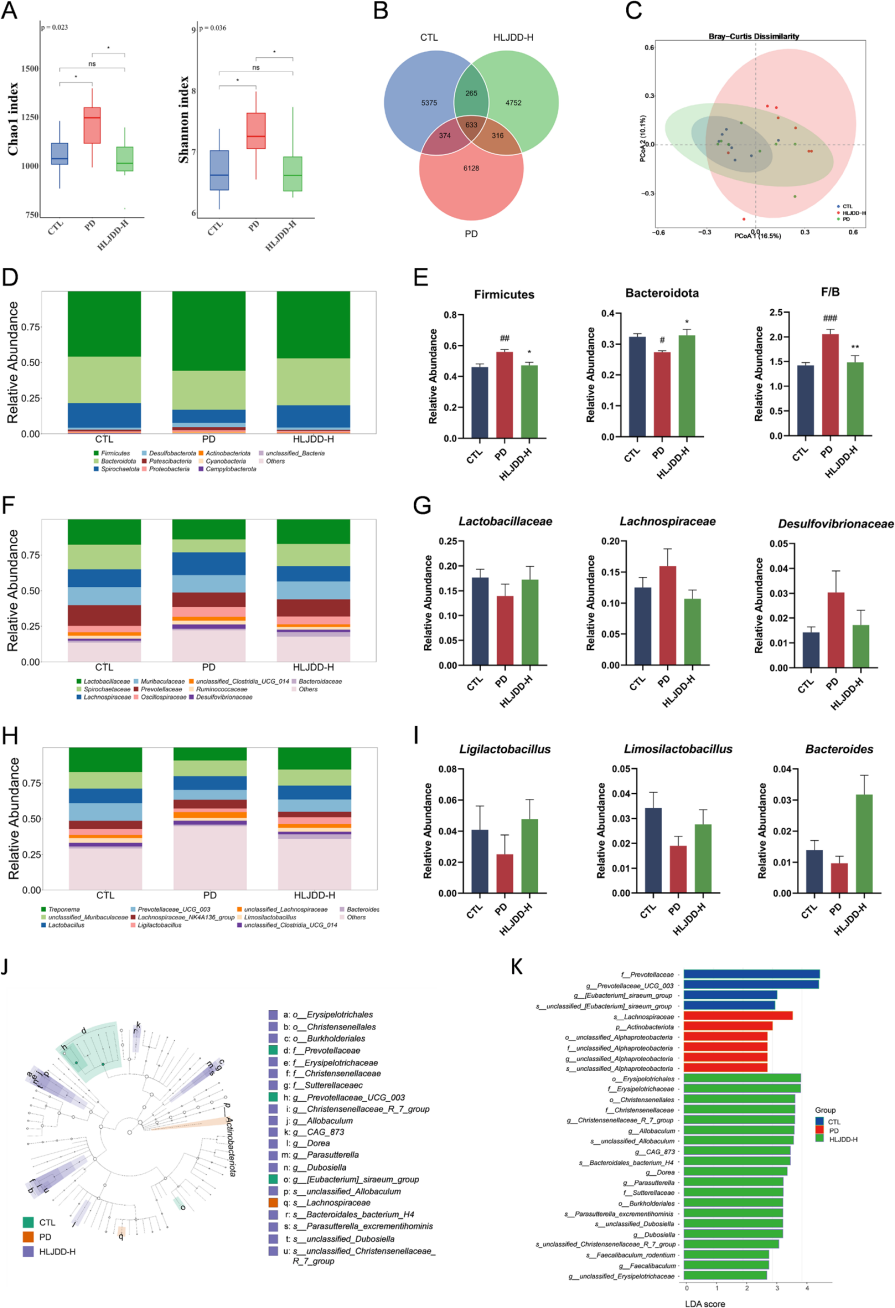
HLJDD treatment modulates the gut microbiota in PD rats. **A** The α-diversity estimated by Chao1 and Shannon index among groups in gut microbiota. **B** A Venn diagram showing the overlaps among groups. **C** Principal coordinate analysis (PCoA) for β-diversity on the basis of Bray–Curtis. CTL vs PD, *r*^2^ = 0.105, *p *= 0.014; H vs P, *r*^2^ = 0.104, *p *= 0.025. **D** Relative abundance of gut microbiota at the phylum level. **E** Relative abundance of *Firmicutes, Bacteroidetes,* and F/B. **F** Relative abundance of gut microbiota at the family level. **G** Relative abundance of *Lactobacillaceae*, *lachnospiraceae*, and *Desulfovibrionaceae* (n = 8). **H** Relative abundance of microbiota at the genus level. **I** Relative abundance of *Ligilactobacillus*, *Limosilactobacillus*, and *Bacteroides* (n = 8). **J**-**K** Cladogram using linear discrimination analysis effect size (LEFSe) analysis of the gut microbiota among groups. Data were expressed as the mean ± SEM. ^#^*P* < 0.05, ^##^*P* < 0.01, ^###^*P* < 0.001, vs. CTL group; ^*^P < 0.05, ^**^P < 0.01, ^***^P < 0.001, vs. PD group

To assess the influence of HLJDD on the gut microbiota composition of SD rats with periodontitis, 16S rRNA sequencing was implemented to specifically target the V3-V4 region of intestinal contents. The intestinal microbiota in the PD group exhibited significantly more diverse and evenness contrasting to the CTL group, as indicated by the Chao1 and Shannon indices of alpha diversity. However, these indices markedly decreased after HLJDD-H treatment (Fig. 5A). A Venn diagram displayed 641 shared operational taxonomic units (OTUs) across groups, along with 6365, 5006, and 4765 unique OTUs in the CTL, PD, and HLJDD-H groups, respectively (Fig. 5B). Principal Coordinate Analysis (PCoA) additionally verified that HLJDD-H treatment altered gut microbiota composition in comparison to the PD group (Fig. 5C).

To evaluate the overall microbial composition alterations, the relative abundance of the top 10 dominant taxa was analyzed. *Firmicutes* and *Bacteroidetes* were the predominant phylum across all groupings (Fig. 5D). Contrary to the CTL group, PD rats demonstrated an increased *Firmicutes/Bacteroidetes* (F/B) ratio due to elevated *Firmicutes* and reduced *Bacteroidetes*. Treatment with HLJDD, however, reversed this trend (*p* < 0.05) (Fig. 5E). The top 10 most common families in each group are shown in Fig. 5F. The PD group had lower abundances of *Lactobacillaceae*, *Spirochaetaceae*, *Muribaculaceae*, *Prevotellaceae*, and *Bacteroidaceae*, alongside increased *lachnospiraceae*, *Oscillospiraceae*, *unclassified_Clostridia_UCG_014*, *Ruminococcaceae* and *Desulfovibrionaceae*, compared to the CTL group. Although HLJDD-H treatment counteracted these changes, they were not statistically relevant (Fig. 5G and Fig. S1A). At the genus level, Fig. 5H illustrates the 10 most prevalent genera across groups. HLJDD-H treatment increased the abundances of *Treponema*, *unclassified_Muribaculaceae*, *Lactobacillus*, *Prevotellaceae_UCG_003*, *Ligilactobacillus*, *Limosilactobacillus*, and *Bacteroides*, while reducing other genera compared to the PD group (Fig. 5I and Fig. S1B). Linear discriminant analysis effect size (LEfSe) showed greater abundances of *Lachnospiraceae* and *Alphaproteobacteria* in the PD group. Conversely, HLJDD treatment increased the abundance of *Erysipelotrichaceae*, *Christensenellaceae*, *CAG_873*, *Allobaculum*, *Bacteroides_bacterium_H4*, *Dorea*, *Parasutterella*, *Sutterellaceae*, *Dubosiella*, and *Faecalibaculum* in PD rats (Fig. 5J–K). Collectively, these findings demonstrate that HLJDD effectively modulates gut microbiota composition.

### HLJDD Relieved intestinal inflammation and repaired the intestinal barrier in periodontitis rats

**Fig. 6:**
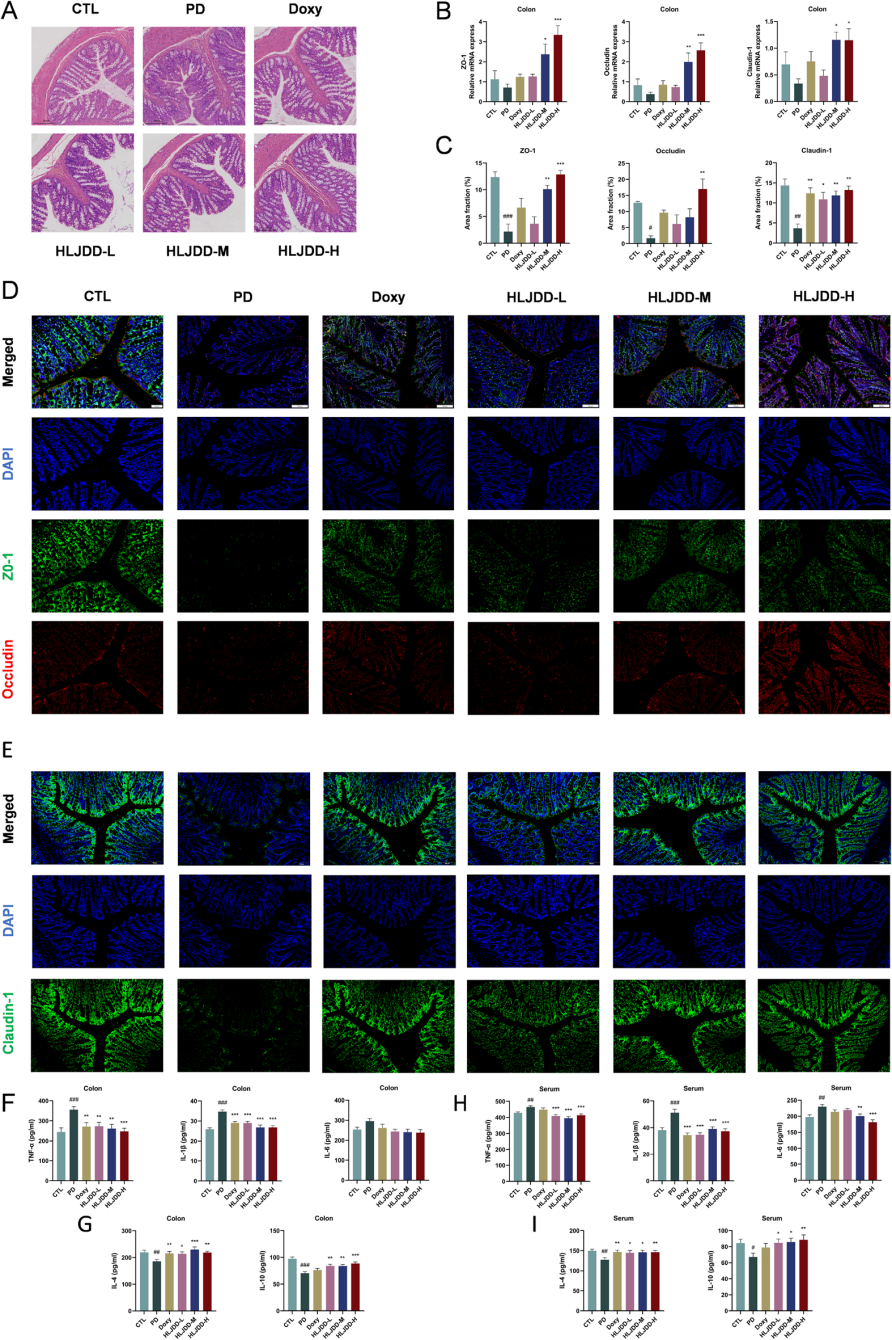
HLJDD restores intestinal barrier function and ameliorate intestinal inflammation in PD rats. **A** Representative images of colon tissue H&E staining. Scale bar = 200 µm. (n = 3) (**B**) Relative mRNA expressions of ZO-1, occludin, and claudin-1 in colon. (n = 6) (**C**) Quantification of area fractions in the colon of rats. (n = 3) (**D**-**E**) Representative images of ZO-1, occludin, and claudin-1 immunostaining in the colon. Scale bar = 100 µm. (n = 3) (**F**-**I**) The ELISA test indicating the levels of TNF-α, IL-1β, IL-6, IL-4, and IL-10 in serum and colon of rats (n = 6). Data were expressed as the mean ± SEM. ^#^*P* < 0.05, ^##^*P* < 0.01, ^###^*P* < 0.001, vs. CTL group; ^*^P < 0.05, ^**^P < 0.01, ^***^P < 0.001, vs. PD group

Recent investigations have demonstrated that disruption of the gut microbiota ecosystem may directly impair intestinal barrier function and trigger chronic inflammatory responses. To study the therapeutic potential of HLJDD, we evaluated its effects on intestinal barrier integrity and local/systemic inflammation in PD rats. H&E staining revealed significant colonic damage in PD rats compared to the CTL group, characterized by reduced goblet cells, crypt architectural distortion, and inflammatory cell infiltration (Fig. 6A). However, both Doxycycline and HLJDD treatment significantly ameliorated these pathological changes. IF analysis demonstrated dramatically reduced expression of tight junction proteins (ZO-1, occludin, and claudin-1) in PD rats, which was dose-dependently restored by HLJDD treatment (Fig. 6C-E). RT-qPCR analysis confirmed these findings at the mRNA expression level (Fig. 6B), indicating HLJDD's ability to enhance intestinal barrier integrity. In comparison to the CTL group, the PD rat colons exhibited substantially increased pro-inflammatory mediators (TNF-α and IL-1β) and diminished anti-inflammatory factors (IL-4 and IL-10) as determined by ELISA. HLJDD treatment effectively reversed these alterations (Fig. 6F–G). Similar cytokine patterns were observed systemically, with serum levels mirroring intestinal changes (Fig. 6H–I). Notably, while IL-6 showed comparable trends, the changes were not statistically significant (Fig. 6F). Furthermore, HLJDD treatment effectively decreased serum LPS levels (Fig. S2). These findings indicate that HLJDD treatment protects intestinal barrier function and modulates both local and systemic inflammatory responses in PD rats.

### Network pharmacological investigation of HLJDD in the management of periodontitis-related cognitive impairment

**Fig. 7:**
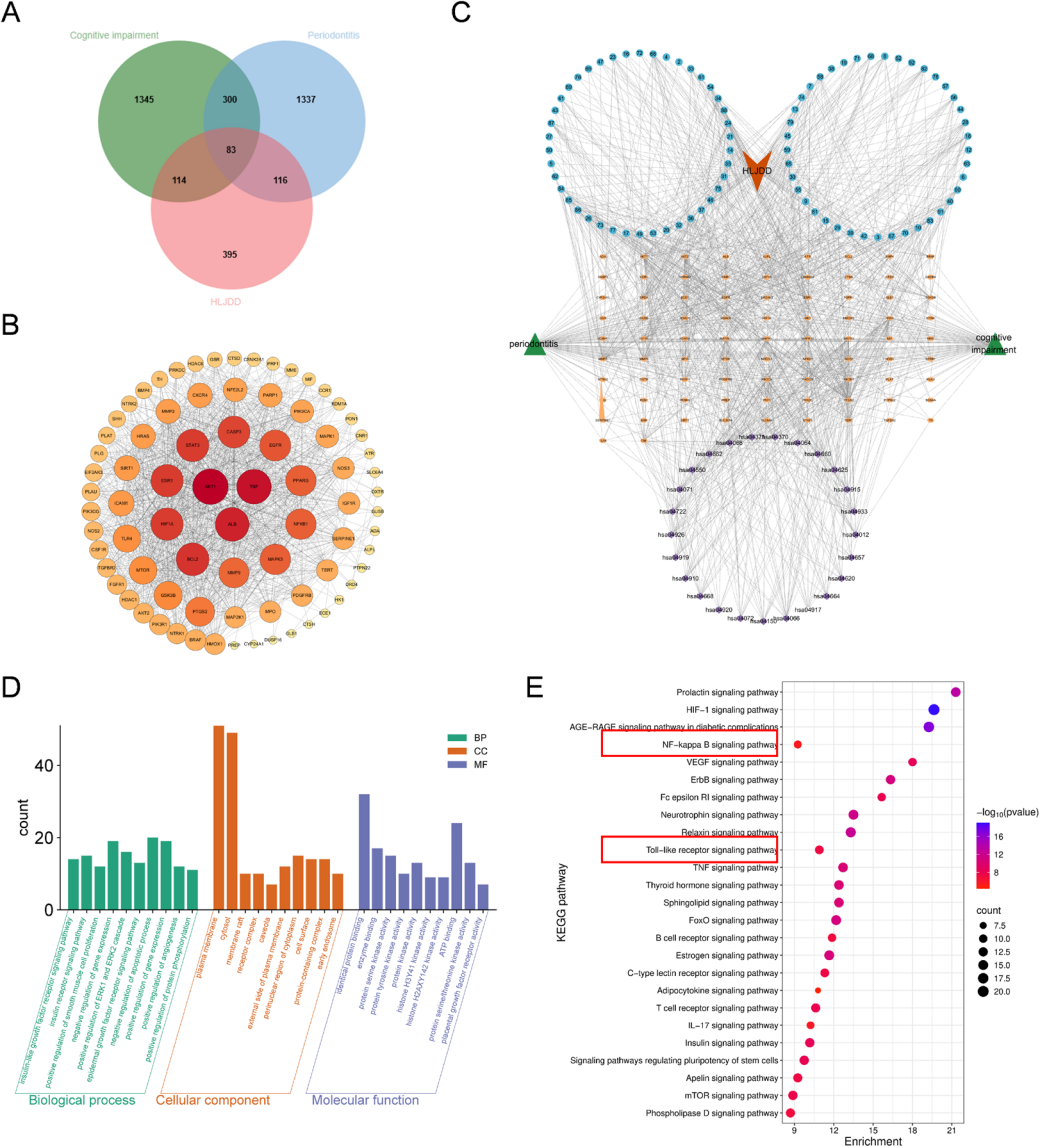
Network pharmacology analysis of HLJDD in the treatment of periodontitis-related cognitive impairment. **A** Venn diagram of the intersection of PD, cognitive disorders, and HLJDD-related genes. **B** PPI network of core targets. **C** HLJDD-compounds-key targets-pathways visualization network. Compounds are denoted by blue, targets by orange and pathways by green. **D** GO enrichment analysis: the top 10 biological processes (green box), cellular components (orange box), and molecular functions (purple box). **E** Bubble chart depicts the top 25 KEGG pathways related to periodontitis-related cognitive disorder

Network pharmacological was adopted to investigate the basic mechanism of HLJDD in periodontitis-related cognitive impairment. 94 potentially active compounds of HLJDD were assembled for target prediction, resulting in the identification of 768 unique compound-related targets after the removal of duplicates. From four databases: DrugBank, GeneCards, OMIM, and TTD, disease-associated targets were identified, including 1840 periodontitis-associated genes and 1876 cognitive disorder-related genes. A Venn diagram analysis revealed 83 overlapping targets among these groups for further investigation (Fig. 7A). Subsequently, the identified targets were imported into the STRING database to construct a protein–protein interaction (PPI) network. The resulting network comprised 83 nodes and 1038 edges, exhibiting an average node degree of 25 (Fig. 7B). Using Cytoscape, a comprehensive compound-target-disease-pathway network was generated (Fig. 7C), demonstrating that multiple HLJDD components may act synergistically on various targets to exert therapeutic effects.

GO enrichment and KEGG studies were conducted to identify potential pathways involved in periodontitis-related cognitive impairment. GO analysis revealed enrichment in 531 biological processes (BPs), 132 molecular functions (MFs), and 60 cellular components (CCs) with the top 10 terms in each category displayed (Fig. 7D). KEGG analysis identified significant enrichment in 145 signaling pathways, among which the top 25 most enriched pathways visualized (Fig. 7E). Notably, key pathways associated with neuroinflammation and cognitive impairment included the Toll-like receptor (TLR) and NF-κB signaling pathways, as well as nervous system development and synaptic function. These findings suggest that HLJDD may alleviate periodontitis-related cognitive disorder by regulating the TLR4/NF-κB signaling pathway.

### HLJDD alleviated BBB damage and inhibited neuroinflammation by TLR4/NF-κB signaling pathway in PD rats

**Fig. 8:**
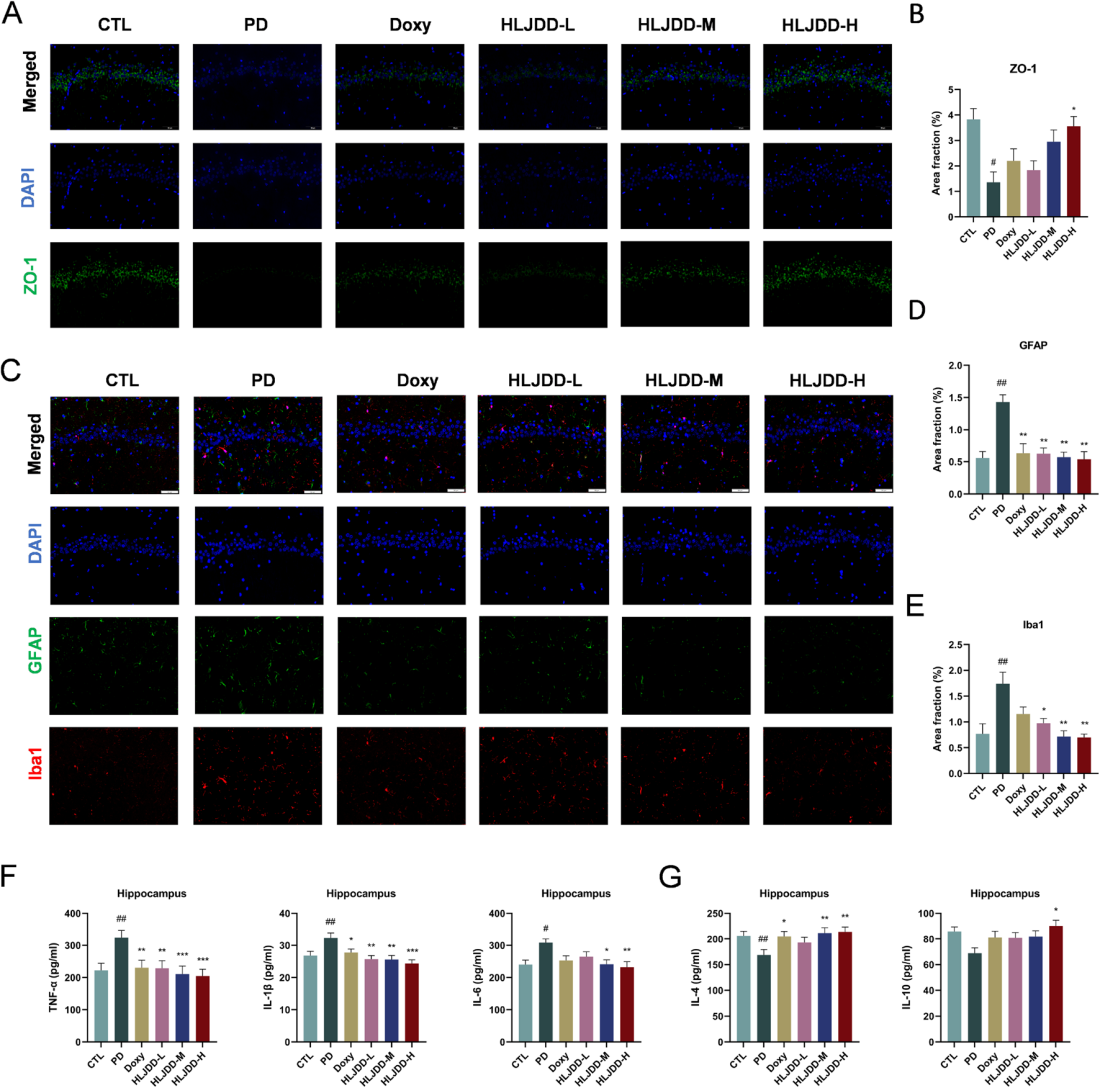
HLJDD alleviated BBB damage and inhibited neuroinflammation in PD rats. **A** Representative images of ZO-1 immunostaining. Scale bar = 50 µm. (n = 3) (**B**) Quantification of area fractions in the hippocampus of rat. (n = 3) (**C**) Representative images of astrogliosis (GFAP) and microgliosis (Iba1) in the hippocampus. Scale bar = 50 µm. (n = 3) (**D**-**E**) Area fractions (%) of GFAP and Iba1 in the hippocampus of rats. (n = 3) (**F**-**G**) The level of TNF-α, IL-1β, IL-6, IL-4 and IL-10 in hippocampus using ELISA detection. (n = 6)

To determine whether HLJDD influences blood–brain barrier (BBB) integrity, we evaluated key tight junction proteins using immunofluorescence (IF) staining. IF analysis revealed significantly decreased ZO-1 protein levels in vehicle-treated PD rats compared to HLJDD-treated PD rats (Fig. 8A, B). Given the crucial role of astrocytes and microglia in neuroinflammation, we quantified their activation markers. PD rats exhibited significantly increased Iba-1 (microglial) and GFAP (astrocytic) immunoreactivity in hippocampal CA1 regions, which was dose-dependently attenuated by both doxycycline and HLJDD (Fig. 8C–E). ELISA analysis demonstrated elevated hippocampal concentrations of pro-inflammatory meditors (TNF-α, IL-1β, and IL-6) coupled with reduced anti-inflammatory factors (IL-4 and IL-10) in PD rats versus controls. HLJDD treatment significantly reversed these alterations (Fig. 8F, G). These findings indicate that HLJDD enhances BBB integrity and mitigates neuroinflammation.

**Fig. 9:**
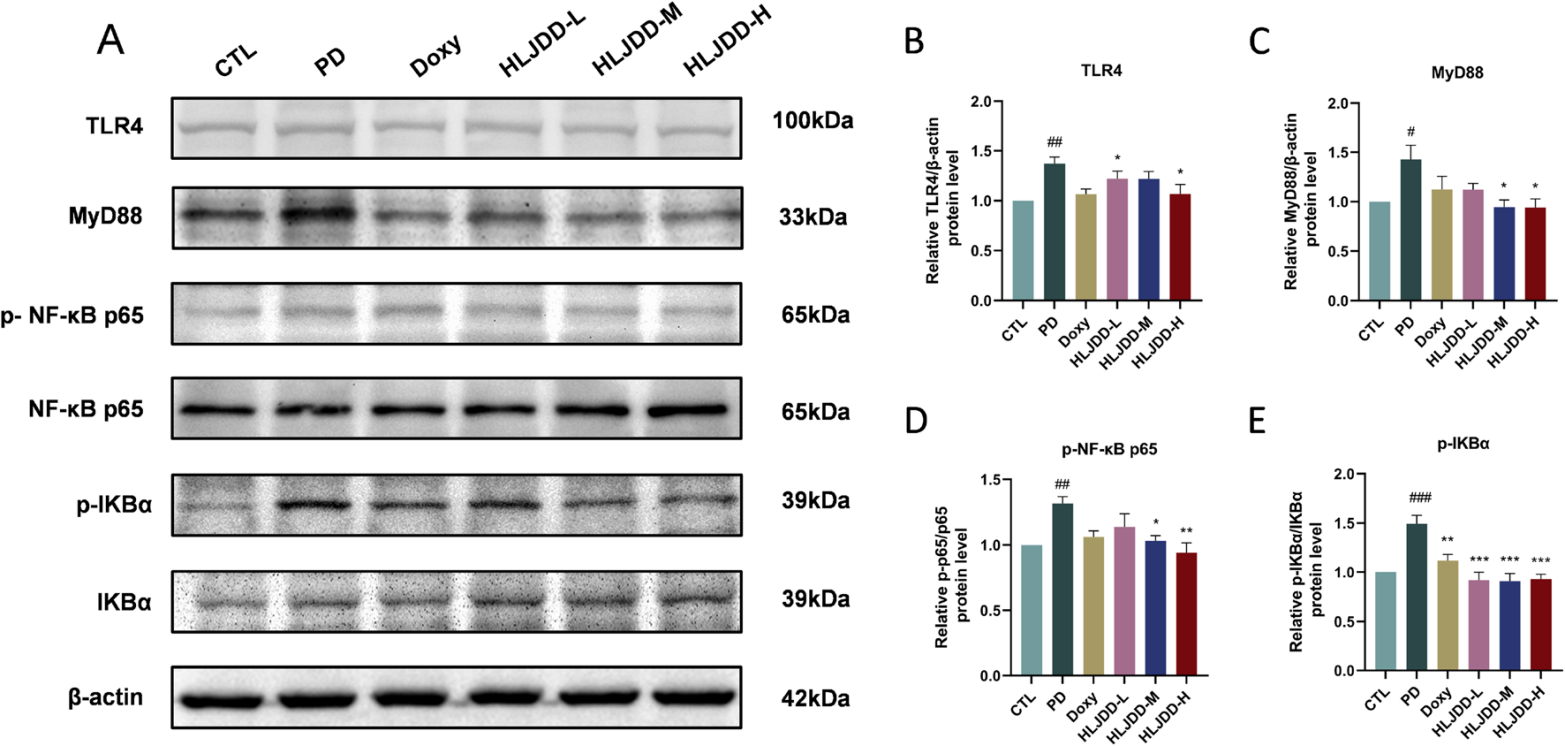
HLJDD treatment inhibited the activation of TLR4 and NF-κB signaling pathways in the hippocampal tissue. **A** Representative immunoblots of TLR4, MyD88, NF-κB-p-p65, and p-IκBα in the hippocampus. (n = 6) (**B**-**E**) Quantitative analysis of the expression of TLR4, MyD88, NF-κB-p-p65, and p-IκBα in the hippocampus. (n = 6) Data were expressed as the mean ± SEM. ^#^*P* < 0.05, ^##^*P* < 0.01, ^###^*P* < 0.001, vs. CTL group; ^*^P < 0.05, ^**^P < 0.01, ^***^P < 0.001, vs. PD group

Based on network pharmacology and prior evidence, we investigated HLJDD's effects on TLR4/NF-κB signaling. Western blot analysis showed upregulated hippocampal expression of p-NF-κB-p65, p-IKBα, TLR4, and MyD88 in PD rats, which HLJDD treatment significantly suppressed (Fig. 9). Collectively, these findings confirmed that HLJDD effectively inhibits TLR4/NF-κB pathway activation in PD rats.

## Discussion

Growing evidence demonstrates a strong association between periodontitis (PD) and cognitive dysfunction [25, 26]. However, therapeutic interventions for PD-induced cognitive impairment remain unexplored. This study investigates the neuroprotective mechanisms of HLJDD against PD-related cognitive impairment via the microbiota-gut-brain axis, building upon established evidence of HLJDD's gut microbiota regulatory properties. We found that the oral administration of HLJDD for a period of 8 weeks dramatically reduced alveolar bone loss, improved cognitive performance, decreased neuronal injury and Aβ deposition, attenuated glial activation, and restored gut microbiota composition in PD rats. Beyond barrier protection, HLJDD exhibited the effects of inhibiting inflammatory responses, significantly downregulating pro-inflammatory markers (TNF-α, IL-1β, IL-6) while upregulating anti-inflammatory factors (IL-4, IL-10) across intestinal tissue, systemic circulation, and central nervous system compartments. Our mechanistic investigations revealed hippocampal suppression of TLR4/MyD88/NF-κB cascade activation. This study provides evidence that HLJDD alleviates PD-associated cognitive impairment primarily through its anti-inflammatory effects and barrier protection, presenting a promising therapeutic strategy for periodontitis-related neurodegeneration.

A well-established method for studying periodontal disease, The *P. gingivalis*-adhered ligature periodontitis model, promotes plaque accumulation and inflammatory cell infiltration around molars, ultimately causing alveolar bone loss and a long-lasting infection of *P. gingivalis* [27]. Previous studies demonstrate that this model induces rapid alveolar bone resorption within two weeks along with sustained periodontal inflammation [28, 29]. In our study, rats received either HLJDD, doxycycline, or vehicle treatment for eight weeks following the two-week ligation period. Our results showed that periodontitis induced by *P. gingivalis*-adhered ligature not only triggered significant periodontal inflammation but also caused substantial alveolar bone loss. Consistent with other studies on PD model [12, 30], we observed PD rats displayed decline in learning and memory ability and neuropathological changes, including substantial neuronal loss, reactive gliosis, and elevated cerebral inflammatory markers. In addition, HLJDD treatment effectively attenuated these periodontal pathologies while also improving cognitive function in the MWM test. Meanwhile, HLJDD showed considerable reduction in neurodegenerative features, including attenuated neuronal degeneration and diminished amyloid-beta deposition compared to PD rats. These collective results suggest the potential therapeutic value of HLJDD address both cognitive decline and neural damage associated with chronic periodontitis.

Initial experimental findings revealed that HLJDD produced therapeutic outcomes in a dose-dependent manner within the periodontitis rat model, where the HLJDD-H group exhibited the most marked improvement. Consequently, this dosage was chosen for further 16S rRNA sequencing to clarify the mechanism through which HLJDD regulate gut microbiota. Emerging evidence indicates that periodontitis may alter gut microbiota composition via the translocation of periodontal pathogen to the intestines, thereby disturbing ecological balance and potentially exacerbating neuroinflammation, Aβ accumulation, and cognitive decline [31, 32]. While microbiota dysbiosis is thought to play a role in this pathway, our 16S rRNA sequencing analysis did not detect significant changes in overall microbiota structure following HLJDD intervention, even though shifts in alpha and beta diversity were noted. These results imply that the central mechanisms underlying HLJDD’s efficacy are likely not rooted in extensive reorganization of the gut microbial community. Rather, our findings emphasize that its beneficial actions are more substantially tied to pronounced anti-inflammatory effects and the enhancement of key physiological barriers.

A critical aspect of cognitive impairment in periodontitis is the disruption of the microbiota-gut-brain axis, particularly through local and systematic inflammation and barrier dysfunction [33]. The disruption of gut microbial balance may compromise intestinal homeostasis, impair barrier integrity, resulting in intestinal inflammation. furthermore, chronic mild gut inflammation could exacerbate cognitive dysfunction through gut-brain interactions[34]. Colonic tight junction proteins protect the intestinal barrier, preventing toxic metabolites or substances like LPS from entering the bloodstream and triggering inflammatory responses [35]. In line with previous reports [33], we found that pathological changes occurred in the colon tissue in the PD rats. Tight junction proteins, which are of great significance for maintaining intestinal barrier integrity, were notably downregulated in PD animals, accompanied by elevated levels of pro-inflammatory cytokines. Conversely, HLJDD administration effectively upregulated tight junction protein expression, enhanced anti-inflammatory cytokine production, and suppressed pro-inflammatory mediators, thereby restoring intestinal barrier function and mitigating inflammation. Considering the established link between gut-derived inflammation, barrier dysfunction, and neural communication [36], we further assessed systemic inflammatory responses and the level of LPS. Our results demonstrated that HLJDD treatment significantly lowered circulating pro-inflammatory cytokine concentrations and the level of LPS. while increasing anti-inflammatory factors. These observations reinforce the concept that gut dysbiosis-induced barrier impairment facilitates the systemic dissemination of harmful metabolites, subsequently promoting inflammatory cascades that may contribute to cognitive impairment.

Inflammatory mediators originating from the gut can enter systemic circulation and subsequently impair BBB, thereby inducing neuroinflammatory processes [37, 38]. Prior research indicates that systemic inflammation may accelerate neurodegeneration, often mediated by a disrupted BBB [39]. Critical to the maintenance of BBB integrity are tight junction proteins, whose decreased expression is a hallmark of barrier impairment. Our results demonstrated that HLJDD treatment significantly increased the expression of central BBB tight junction components, including ZO-1, indicating a reparative influence on barrier function. Additionally, neuroinflammation primarily arises from the activation of microglial and astrocytic cells, leading to the secretion of various cytokines and chemokines [40, 41] Our data show that HLJDD administration suppressed the activation of Iba-1 and GFAP in hippocampal regions. These changes were accompanied by a marked decrease in hippocampal levels of pro-inflammatory cytokines (TNF-α, IL-1β, IL-6) and an increase in anti-inflammatory factors (IL-4, IL-10), indicating a potent suppression of neuroinflammatory responses. The concomitant reduction in Aβ deposition further supports the neuroprotective effects of HLJDD. Collectively, these findings imply that HLJDD mitigates the deleterious cycle of inflammatory propagation and barrier failure within the gut–brain axis.

The findings from network pharmacology and earlier studies suggest that the active ingredients in HLJDD work together to help with periodontitis-related cognitive impairment through blocking TLR4/NF-κB activation [18]. Activation of this pathway, often triggered by systemic inflammatory signals like LPS, leads to the production of inflammatory cytokines that exacerbate cognitive impairment [30, 42]. Critical to this process, MyD88 serves as the central adaptor protein linking TLR4 activation to subsequent NF-κB phosphorylation events [43]. The phosphorylated NF-κB transcription factor translocate to the nucleus, which subsequently triggers the synthesis of inflammatory factors [44]. In PD rats, HLJDD was found to reduce the protein expression of TLR4, MyD88, p-NF-κB p65, and p-IKBα. However, HLJDD treatment significantly inhibited the hippocampal expression of TLR4, MyD88, p-IKBα, and p-NF-κB p65. Thus, HLJDD’s neuroprotective effects are likely mediated through its direct anti-inflammatory action, by quenching the TLR4/NF-κB signaling cascade, thereby protecting barrier integrity and reducing inflammation throughout the gut-brain axis.

## Conclusion

In conclusion, our findings demonstrate that HLJDD ameliorates cognitive impairment in periodontal model rats. The proposed mechanism of action prioritizes the reduction of neuroinflammation and the preservation of intestinal and blood–brain barrier integrity, with the inhibition of the TLR4/NF-κB signaling pathway playing a central role. While a role for gut microbiota cannot be entirely ruled out, our data did not show significant microbial shifts with HLJDD treatment, suggesting its effects are more directly linked to its anti-inflammatory and barrier-protective properties. This study proposes HLJDD as a promising multi-target therapeutic agent against periodontitis-associated cognitive impairment by targeting critical inflammatory and barrier pathways along the microbiota-gut-brain axis. Further research is warranted to validate these mechanisms and explore clinical translation.

## Supplementary Information

Supplementary file 1.

Supplementary file 2.

## Data Availability

On request, the data will be made accessible to the user.

## Abbreviations

Aβ: β-amyloid
AB: alveolar bone
ABC: alveolar bone crest (ABC)
AD: Alzheimer’s disease
BBB: blood-brain barrier
CA1: cornu ammonis 1
CA3: cornu ammonis 3
CEJ: cemento-enamel junction
CTL: control
DG: dentate gyrus
Doxy: doxycycline
HLJDD: Huang-Lian-Jie-Du decoction
HLJDD-H: HLJDD high-dose group
HLJDD-L: HLJDD low-dose group
HLJDD-M: HLJDD medium-dose group
IF: immunofluorescence
IHC: immunohistochemistry
LPS: Lipopolysaccharide
micro-CT: micro-computed tomography
MWM: Morris water maze
OTUs: operational taxonomic units
PD: periodontitis
P. *gingivalis*: *Porphyromonas gingivalis*
SD: Sprague-Dawley
SPF: specific pathogen-free
UHPLC-Q-Orbitrap HRMS: ultra-high performance liquid chromatography coupled with quadrupole-Orbitrap high-resolution mass spectrometry
