# Supplementary material for: Huang-Lian-Jie-Du decoction alleviates cognitive impairment in periodontitis rats through restoring microbiota-gut-brain axis and inhibiting neuroinflammation via TLR4/NF-κB pathway
Source: Chin Med. 2025 Oct 23;20:179. doi: 10.1186/s13020-025-01235-6 (PMC12548159; doi:10.1186/s13020-025-01235-6)
Supplement: Supplementary file 1 [file 13020_2025_1235_MOESM1_ESM.docx]

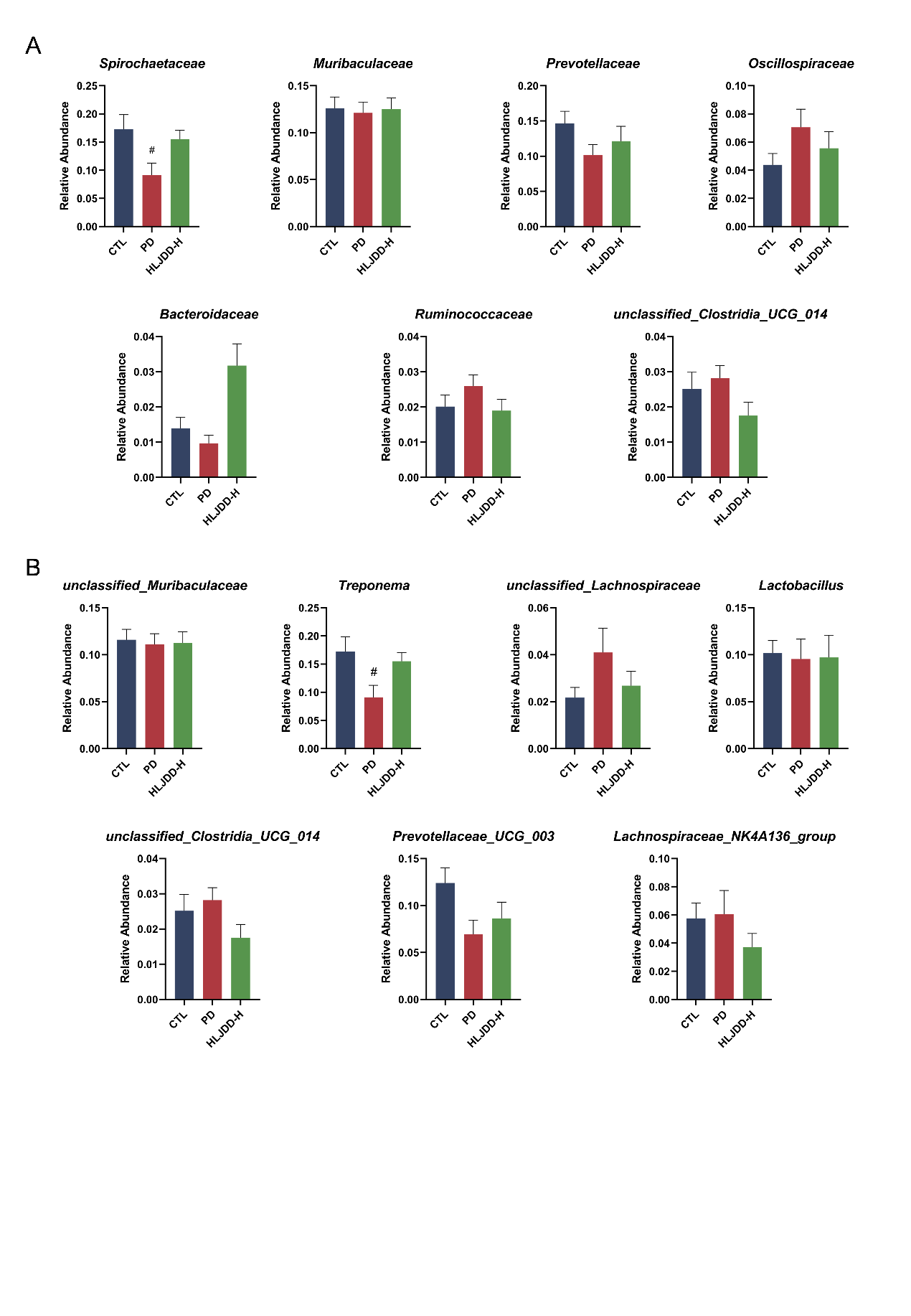


Fig S1(A) Relative abundance of *Spirochaetaceae*, *Muribaculaceae*, *Prevotellaceae*, *Oscillospiraceae*, *Bacteroidaceae*, *Ruminococcaceae*, and *unclassified_Clostridia_UCG_014*. (n=8) (B) Relative abundance of *unclassified_Muribaculaceae*, *Treponema*, *unclassified_ Lachnospiraceae, Lactobacillus*, *unclassified_Clostridia_UCG_014*, *Prevotellaceae_UCG_003*, *Lachnospiraceae_NK4A136_group.*(n=8)


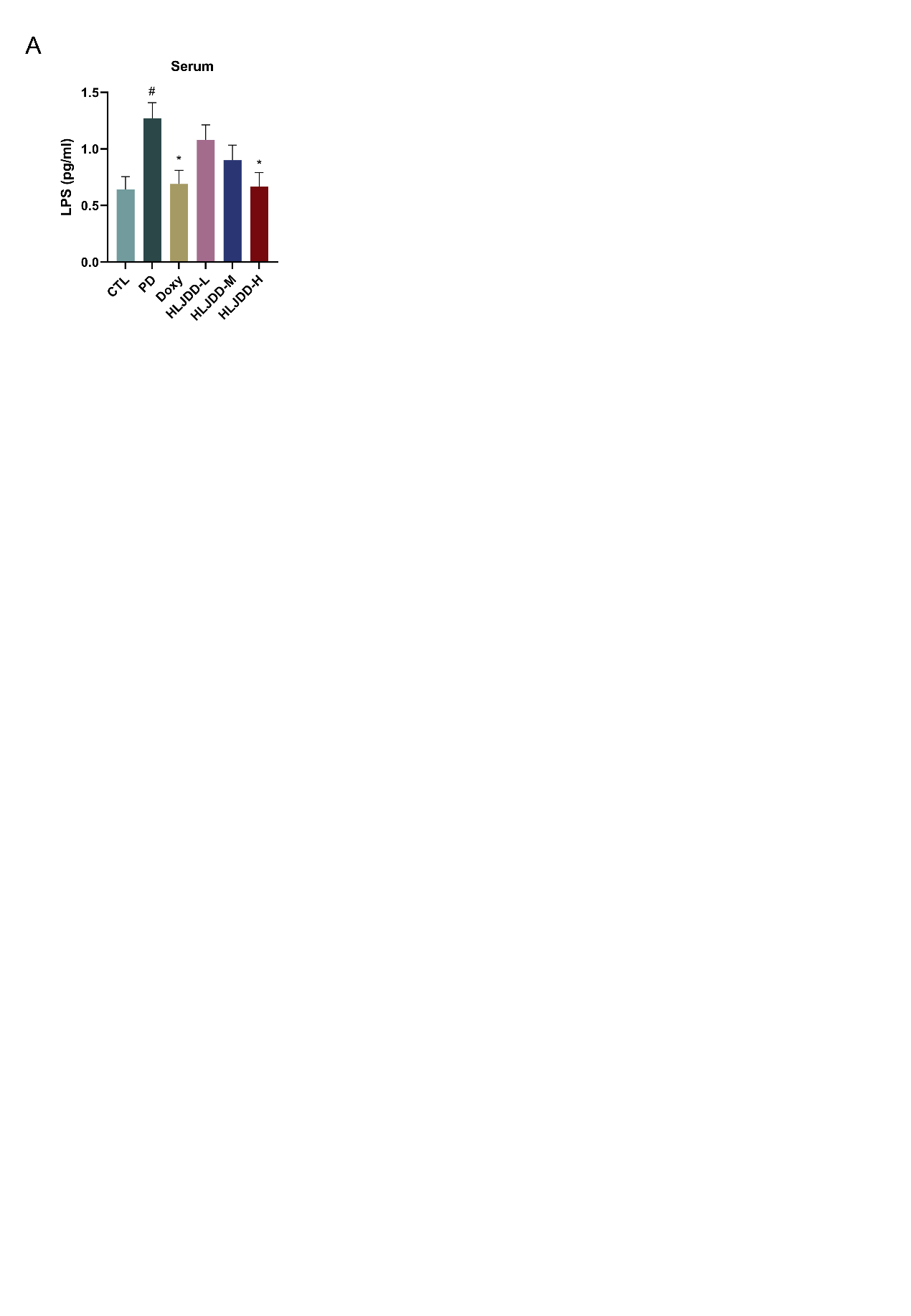


Fig S2(A)The levels of LPS in serum using Elisa. (n=8)
