# Supplementary material for: Huang-Lian-Jie-Du decoction alleviates cognitive impairment in periodontitis rats through restoring microbiota-gut-brain axis and inhibiting neuroinflammation via TLR4/NF-κB pathway
Source: Chin Med. 2025 Oct 23;20:179. doi: 10.1186/s13020-025-01235-6 (PMC12548159; doi:10.1186/s13020-025-01235-6)
Supplement: Supplementary file 2 [file 13020_2025_1235_MOESM2_ESM.docx]

**Table S1** The chemical composition of HLJDD by UHPLC-Q-Exactive Orbitrap HRMS

| **No.** | **Formula** | **tR/**  **min** | **Theoretical**  **(Da)** | **Calculated**  **mass (Da)** | **Error**  **(ppm)** | **Adducts** | **MS Fragmentation** | **Component name** | **Classification** |
| --- | --- | --- | --- | --- | --- | --- | --- | --- | --- |
| 1 | C_6_H_14_O_6_ | 0.7 | 227.0772 | 227.0762 | -4.76 | [M-H]^-^ | 89.0229, 101.0230, 181.0704, 227.0221 | DL-Mannitol | Carbohydrates and Glycosides |
| 2 | C_7_H_6_O_4_ | 6.88 | 153.0193 | 153.018 | -8.69 | [M-H]^-^ | 109.0281, 153.0180 | Protocatechuic acid | Others |
| 3 | C_16_H_22_O_10_ | 7.72 | 373.114 | 373.1131 | -2.52 | [M-H]^-^ | 59.0124, 71.0125, 89.0230, 119.0336,123.0438,149.0593, 167.0701, 179.0550, 193.0496, 373.1130 | Gardoside | Terpenes |
| 4 | C_16_H_24_O_11_ | 8.46 | 391.1246 | 391.1238 | -1.94 | [M-H]^-^ | 59.0124, 89.0230, 167.0700, 185.0807, 229.0707, 391.1237, 392.1271 | Shanzhiside | Terpenes |
| 5 | C_16_H_22_O_10_ | 8.62 | 373.114 | 373.1131 | -2.52 | [M-H]^-^ | 59.0125, 71.0126, 89.0231, 123.0439, 149.0596, 153.0544, 167.0703, 211.0605, 315.1077, 373.1134 | Geniposidic acid | Terpenes |
| 6 | C_17_H_24_O_11_ | 9.09 | 449.1301 | 449.1293 | -1.58 | [M-H]^-^ | 101.0231, 139.0389, 179.0552, 241.0713, 403.1242, 449.1298 | Methyl deacetylasperulosidate | Terpenes |
| 7 | C_16_H_18_O_9_ | 9.15 | 353.0878 | 353.0871 | -2.1 | [M-H]^-^ | 135.0440, 179.0341, 191.0553, 353.0876 | Neochlorogenic acid | Phenylpropanoids |
| 8 | C_17_H_24_O_11_ | 9.56 | 427.1211 | 427.1204 | -1.69 | [M+H]^+^ | 427.1207 | Gardenoside | Terpenes |
| 9 | C_16_H_24_O_10_ | 9.69 | 375.1297 | 375.1287 | -2.51 | [M-H]^-^ | 59.0125, 89.0231, 107.0490, 125.0595, 151.0753, 169.0859, 213.0761, 375.1291, 376.1326 | Mussaenosidic acid | Terpenes |
| 10 | C_16_H_17_NO_3_ | 9.96 | 272.1281 | 272.1279 | -0.99 | [M+H]^+^ | 107.0495, 161.0597, 255.1013, 272.1277 | Higenamine | Organoheterocyclic compounds |
| 11 | C_17_H_24_O_11_ | 10.15 | 449.1301 | 449.1292 | -1.89 | [M-H]^-^ | 101.0231, 139.0388, 241.0712, 403.1240, 449.1295 | Feretoside | Lipids and lipid-like molecules |
| 12 | C_16_H_26_O_8_ | 10.86 | 391.161 | 391.1603 | -1.74 | [M-H]^-^ | 71.0124, 89.0229, 101.0229, 113.0229, 161.0441, 165.0906, 179.0547, 315.1438, 327.1438, 391.1597 | Jasminoside B | Terpenes |
| 13 | C_16_H_22_O_8_ | 10.96 | 365.1207 | 365.1202 | -1.26 | [M+H]^+^ | 365.1197, 366.1227 | Coniferin | Carbohydrates and Glycosides |
| 14 | C_16_H_20_O_9_ | 11.11 | 379.1 | 379.0991 | -2.22 | [M+H]^+^ | 379.0997, 380.1023 | Trans-ferulic acid-4-beta-glucoside | Carbohydrates and Glycosides |
| 15 | C_16_H_18_O_9_ | 11.39 | 353.0878 | 353.0869 | -2.58 | [M-H]^-^ | 191.0549, 353.0868 | Chlorogenic acid | Phenylpropanoids |
| 16 | C_16_H_18_O_9_ | 11.85 | 353.0878 | 353.0871 | -2.12 | [M-H]^-^ | 93.0332, 135.0437, 173.0443, 179.0337, 191.0549, 353.0869 | Cryptochlorogenic acid | Phenylpropanoids |
| 17 | C_9_H_8_O_4_ | 11.87 | 181.0495 | 181.0493 | -1.16 | [M+H]^+^ | 135.0441, 145.0284, 163.0388 | Caffeic acid | Phenylpropanoids |
| 18 | C_17_H_20_O_9_ | 11.89 | 367.1035 | 367.1023 | -3.13 | [M-H]^-^ | 134.0361, 193.0497, 367.1028 | 5-Feruloylquinic acid | Phenylpropanoids |
| 19 | C_23_H_34_O_15_ | 12.15 | 595.188 | 595.1871 | -1.48 | [M-H]^-^ | 68.9968, 101.0231, 123.0439, 207.0655, 225.0762, 549.1823, 595.1874 | Genipin 1-beta-D-gentiobioside | Terpenes |
| 20 | C_16_H_20_O_9_ | 12.88 | 401.1089 | 401.108 | -2.22 | [M-H]^-^ | 178.0261, 193.0497, 269.1030, 355.1031, 401.1479 | Cis-Ferulic acid 4-O-beta-D-glucopyranoside | Carbohydrates and Glycosides |
| 21 | C_20_H_24_NO_4_^+^ | 13.06 | 342.17 | 342.1693 | -1.99 | [M]+ | 178.0859, 192.1015, 342.0942, 342.1694, 343.1725 | Phellodendrine | Alkaloids |
| 22 | C_17_H_24_O_10_ | 13.27 | 406.1708 | 406.1703 | -1.03 | [M+H]^+^ | 121.0649, 149.0596, 159.0438, 177.0544, 181.0856, 191.0701, 209.0806, 227.0911 | Geniposide | Terpenes |
| 23 | C_16_H_18_O_8_ | 13.46 | 361.0894 | 361.0888 | -1.69 | [M+H]^+^ | 361.0887, 362.0919 | 5-O-(E)-p-Coumaroylquinic acid |  |
| 24 | C_19_H_21_NO_4_ | 13.61 | 326.1398 | 326.1393 | -1.59 | [M-H]^-^ |  | Boldine | Alkaloids |
| 25 | C_20_H_24_NO_4_^+^ | 14.42 | 342.17 | 342.1695 | -1.32 | [M+H]^+^ | 58.0658, 192.1015, 205.1094, 265.0854, 297.1113, 342.1693, 343.1724 | Magnoflorine | Alkaloids |
| 26 | C_16_H_26_O_7_ | 14.54 | 353.1571 | 353.1567 | -1.19 | [M+H]^+^ | 353.1562, 354.1596 | Picrocrocin | Terpenes |
| 27 | C_17_H_20_O_9_ | 14.58 | 367.1035 | 367.1024 | -3 | [M-H]^-^ | 93.0332, 134.0360, 173.0444, 191.0551, 193.0494, 367.1026 | 4-Feruloylquinic acid | Phenylpropanoids |
| 28 | C_32_H_42_O_16_ | 15.28 | 727.2455 | 727.2452 | -0.4 | [M-H]^-^ | 151.0386, 357.1334, 519.1860, 681.2388 | Pinoresinol Diglucoside | Phenylpropanoids |
| 29 | C_15_H_10_O_7_ | 15.72 | 303.0499 | 303.0497 | -0.89 | [M+H]^+^ | 303.0491, 304.0525 | Viscidulin I | Flavonoids |
| 30 | C_10_H_10_O_4_ | 15.9 | 193.0506 | 193.0495 | -6.06 | [M-H]^-^ | 134.0360, 137.0230, 139.0389, 149.0595, 178.0260, 193.0496 | (E)-Ferulic acid | Phenylpropanoids |
| 31 | C_9_H_6_O_4_ | 16.52 | 177.0193 | 177.0181 | -7 | [M-H]^-^ | 177.0182 | 5,7-Dihydroxychromone | Flavonoids |
| 32 | C_27_H_30_O_15_ | 17.13 | 593.1512 | 593.151 | -0.32 | [M-H]^-^ | 208.3988, 255.0288, 284.0319, 285.0397, 305.2795, 593.1497 | Kaempferol 3-neohesperidoside | Flavonoids |
| 33 | C_27_H_30_O_16_ | 17.14 | 609.1461 | 609.1458 | -0.59 | [M-H]^-^ | 300.0268, 301.0346, 609.1451 | Rutin | Flavonoids |
| 34 | C_21_H_20_O_12_ | 17.17 | 465.1028 | 465.1024 | -0.75 | [M+H]^+^ | 303.0496, 338.1380 | Isoquercetin | Phenols |
| 35 | C_26_H_28_O_13_ | 17.38 | 549.1603 | 549.1601 | -0.35 | [M+H]^+^ | 309.0749, 363.0856, 375.0857, 393.0961, 411.1066, 465.1173, 495.1279, 513.1387, 531.1492, 549.1596 | Chrysin 6-C-arabinoside 8-C-glucoside | Flavonoids |
| 36 | C_21_H_20_O_12_ | 17.52 | 463.0882 | 463.0877 | -1.06 | [M-H]^-^ | 271.0247, 300.0271, 301.0348, 463.0875 | Isoquercitrin | Flavonoids |
| 37 | C_21_H_18_O_12_ | 17.56 | 461.0726 | 461.0719 | -1.41 | [M-H]^-^ | 113.0229, 175.0235, 285.0396, 461.0710 | Scutellarin | Flavonoids |
| 38 | C_19_H_16_NO_4_^+^ | 17.74 | 322.1074 | 322.1068 | -1.86 | [M]^+^ | 307.0832, 322.1066, 323.1095 | Groenlandicine | Organoheterocyclic compounds |
| 39 | C_20_H_23_NO_4_ | 17.99 | 342.17 | 342.1694 | -1.78 | [M+H]^+^ | 58.0658, 192.1015, 265.0857, 297.1115, 325.1059, 342.1693, 343.1726 | Corypalmine | Alkaloids |
| 40 | C_27_H_30_O_15_ | 18.01 | 593.1512 | 593.1508 | -0.61 | [M-H]^-^ | 181.0493, 205.0497, 207.0282, 208.4152, 223.0592, 284.0317, 285.0385, 305.2513, 549.1569, 593.1507 | Biorobin | Flavonoids |
| 41 | C_26_H_28_O_13_ | 18.26 | 549.1603 | 549.1605 | 0.38 | [M+H]^+^ | 309.0751, 363.0859, 381.0962, 429.1175, 441.1173, 453.1174, 495.1284, 513.1390, 531.1494, 549.1599 | Chrysin 6-C-glucoside 8-C-arabinoside | Flavonoids |
| 42 | C_10_H_8_O_3_ | 18.34 | 177.0546 | 177.0546 | 0.11 | [M+H]^+^ | 105.0702, 145.0284, 149.0596, 159.0439, 177.0545 | 4-Methylumbelliferone | Phenylpropanoids |
| 43 | C_19_H_18_NO_4_^+^ | 18.41 | 324.123 | 324.1228 | -0.68 | [M]^+^ | 309.0987, 324.1222, 325.1282 | Demethyleneberberine | Alkaloids |
| 44 | C_11_H_12_O_4_ | 18.49 | 207.0663 | 207.0653 | -4.64 | [M-H]^-^ | 103.9190, 177.0182, 192.0419, 207.0656 | Sinapaldehyde | Phenylpropanoids |
| 45 | C_27_H_30_O_15_ | 18.72 | 593.1512 | 593.1503 | -1.57 | [M-H]^-^ | 255.0289, 284.0323, 285.0401, 593.1508 | Nicotiflorin | Flavonoids |
| 46 | C_26_H_32_O_11_ | 18.89 | 543.1837 | 543.1832 | -0.83 | [M]^+^ | 543.1845 | Pinoresinol 4-O-beta-D-glucopyranoside | Phenylpropanoids |
| 47 | C_29_H_36_O_15_ | 19.11 | 623.1981 | 623.1975 | -1.04 | [M-H]^-^ | 113.0230, 135.0438, 161.0231, 461.1647, 623.1976 | Isoacteoside | Carbohydrates and Glycosides |
| 48 | C_27_H_30_O_15_ | 19.38 | 595.1658 | 595.1653 | -0.72 | [M+H]^+^ |  | Oroxin B | Flavonoids |
| 49 | C_26_H_32_O_11_ | 19.6 | 538.2283 | 538.2279 | -0.69 | [M]^+^ | 137.0598, 175.0752, 187.0752, 189.0912, 205.0857, 207.7939, 235.0961, 305.1896, 323.1271, 341.1375 | Epipinoresinol-4-O-beta-D-glucoside | Phenylpropanoids |
| 50 | C_19_H_14_NO_4_^+^ | 19.71 | 320.0917 | 320.0913 | -1.41 | [M]^+^ | 320.0909, 321.0939 | Pseudocoptisine | Alkaloids |
| 51 | C_20_H_20_NO_4_^+^ | 19.76 | 338.1387 | 338.1366 | -6.18 | [M]^+^ | 294.1119, 322.1064, 323.1144, 338.1370, 339.1407 | Columbamine | Alkaloids |
| 52 | C_25_H_24_O_12_ | 19.89 | 515.1195 | 515.1184 | -2.06 | [M-H]^-^ | 135.0439, 173.0443, 179.0337, 191.0550, 207.9503, 353.0869, 515.1180 | Isochlorogenic acid C | Phenylpropanoids |
| 53 | C_20_H_18_NO_4_^+^ | 20.08 | 336.123 | 336.1227 | -1.1 | [M]^+^ | 320.0908, 336.1220 | Epiberberine | Alkaloids |
| 54 | C_15_H_10_O_6_ | 20.1 | 285.0405 | 285.0399 | -1.96 | [M-H]^-^ |  | Fisetin | Flavonoids |
| 55 | C_22_H_20_O_12_ | 20.18 | 477.1028 | 477.1025 | -0.57 | [M+H]^+^ | 286.0464, 301.0698, 477.1019 | DiosMetin 7-O-beta-D-Glucuronide | Flavonoids |
| 56 | C_19_H_16_NO_4_^+^ | 20.29 | 322.1074 | 322.1072 | -0.62 | [M]^+^ | 307.0833, 322.1065, 323.1096 | Berberrubine | Alkaloids |
| 57 | C_17_H_14_O_8_ | 20.39 | 347.0761 | 347.0763 | 0.52 | [M+H]^+^ | 314.0417, 332.0521, 347.0756, 348.0787 | Viscidulin III | Flavonoids |
| 58 | C_15_H_10_O_6_ | 20.48 | 285.0405 | 285.0395 | -3.33 | [M-H]^-^ | 285.0403 | Isoscutellarein | Flavonoids |
| 59 | C_21_H_20_O_10_ | 20.52 | 433.1129 | 433.1127 | -0.48 | [M+H]^+^ | 271.0595, 433.1104 | Oroxin A | Flavonoids |
| 60 | C_21_H_18_O_11_ | 20.55 | 447.0922 | 447.0916 | -1.34 | [M+H]^+^ | 271.0594, 447.0910 | Baicalin | Flavonoids |
| 61 | C_20_H_18_NO_4_^+^ | 20.56 | 336.1236 | 336.1225 | -3.21 | [M]^+^ | 292.0962,320.0912 | Berberine | Alkaloids |
| 62 | C_21_H_22_NO_4_^+^ | 20.61 | 352.1543 | 352.1538 | -1.59 | [M]^+^ | 308.1276, 322.1068, 336.1224, 337.1301, 352.1536 | Palmatine | Alkaloids |
| 63 | C_21_H_20_O_11_ | 20.71 | 447.0933 | 447.0925 | -1.86 | [M-H]^-^ | 85.0282, 113.0231, 175.0238, 243.0658, 271.0609, 447.0927 | Dihydrobaicalin | Flavonoids |
| 64 | C_21_H_18_O_11_ | 20.84 | 891.1626 | 891.1616 | -1.09 | [M-H]^-^ | 113.0230, 269.0448, 445.0765 | Glychionide A | Flavonoids |
| 65 | C_15_H_12_O_6_ | 20.84 | 269.045 | 269.0442 | -3.16 | [M-H]^-^ | 269.0453 | (±)-Eriodictyol | Flavonoids |
| 66 | C_15_H_10_O_5_ | 20.85 | 271.0601 | 271.0587 | -5.2 | [M+H]^+^ | 271.0595 | 3,7,4'-Trihydroxyflavone | Flavonoids |
| 67 | C_21_H_20_NO_4_^+^ | 20.99 | 350.1387 | 350.1387 | -0.03 | [M]^+^ | 306.1119, 334.1069, 335.1145, 350.1383, 351.1412 | 13-Methylberberine | Alkaloids |
| 68 | C_21_H_18_NO_4_^+^ | 21.01 | 348.123 | 348.1231 | 0.14 | [M]^+^ |  | Chelerythrine | Alkaloids |
| 69 | C_21_H_18_O_10_ | 21.05 | 429.0827 | 429.0817 | -2.31 | [M-H]^-^ | 59.0125, 85.0282, 99.0075, 113.0232, 175.0238, 253.0502, 429.0821 | Chrysin-7-O-glucuronide | Flavonoids |
| 70 | C_22_H_20_O_11_ | 21.05 | 461.1078 | 461.1077 | -0.24 | [M+H]^+^ | 270.0519, 285.0753, 461.1072 | Oroxylin A-7-O-glucuronide | Flavonoids |
| 71 | C_22_H_20_O_12_ | 21.12 | 477.1028 | 477.1024 | -0.73 | [M+H]^+^ | 286.0466, 301.0700, 477.1021 | 5,8-Dihydroxy-6,-methoxyflavone-7-O-glucuronide | Flavonoids |
| 72 | C_21_H_28_O_6_ | 21.19 | 399.1778 | 399.1774 | -1 | [M]^+^ | 399.1776, 400.1819 | Octahydrocurcumin | Phenols |
| 73 | C_22_H_20_O_11_ | 21.28 | 461.1078 | 461.1073 | -1.11 | [M+H]^+^ | 270.0516, 285.0751, 461.1070 | Wogonoside | Flavonoids |
| 74 | C_22_H_20_O_11_ | 21.3 | 499.0637 | 499.0628 | -1.86 | [M]^+^ | 291.0255, 323.0552, 499.0645, 500.0660 | Baicalin methyl ester | Flavonoids |
| 75 | C_23_H_22_O_12_ | 21.42 | 491.1184 | 491.1184 | -0.02 | [M+H]^+^ | 285.0384, 300.0622, 315.0855, 491.1176 | 5,7-Dihydroxy-6,8-dimethoxyflavone-7-O-glucuronide | Flavonoids |
| 76 | C_15_H_10_O_7_ | 21.43 | 283.0243 | 283.0242 | -0.07 | [M-H]^-^ |  | Quercetin | Flavonoids |
| 77 | C_15_H_10_O_5_ | 21.9 | 269.0456 | 269.0451 | -1.64 | [M-H]^-^ | 268.7389, 269.0453 | Apigenin | Flavonoids |
| 78 | C_15_H_10_O_6_ | 21.98 | 285.0405 | 285.0398 | -2.28 | [M-H]^-^ | 125.0230, 151.0023, 285.0393 | Kaempferol | Flavonoids |
| 79 | C_14_H_8_N_2_O | 22.01 | 221.0709 | 221.0709 | -0.14 | [M+H]^+^ | 221.0708 | Canthin-6-one | Alkaloids |
| 80 | C_16_H_14_O_4_ | 22.33 | 271.0965 | 271.0964 | -0.3 | [M+H]^+^ | 167.0337, 271.0598, 271.0961 | Alpinetin | Flavonoids |
| 81 | C_14_H_13_NO_4_ | 22.4 | 260.0917 | 260.0916 | -0.38 | [M+H]^+^ | 227.0572, 245.0678, 260.0913 | Skimmianine | Organoheterocyclic compounds |
| 82 | C_23_H_22_O_11_ | 22.46 | 475.1235 | 475.1233 | -0.38 | [M+H]^+^ | 270.0515, 285.0750, 475.1234 | Oroxylin A 7-O-beta-D-glucuronide methyl ester | Flavonoids |
| 83 | C_13_H_11_NO_3_ | 22.54 | 230.0812 | 230.081 | -0.7 | [M+H]^+^ | 215.0573, 230.0808 | gamma-Fagarine | Organoheterocyclic compounds |
| 84 | C_15_H_10_O_5_ | 22.67 | 271.0601 | 271.0598 | -1.11 | [M+H]^+^ | 271.0597 | Norwogonin | Flavonoids |
| 85 | C_32_H_44_O_14_ | 24.57 | 697.2713 | 697.2708 | -0.69 | [M-H]^-^ | 51.7721, 66.7222, 137.5923, 202.5985, 215.1248, 221.9853, 327.1601, 424.3442, 489.2160, 687.2603 | Crocin IV | Terpenes |
| 86 | C_20_H_17_NO_5_ | 24.83 | 374.0999 | 374.0993 | -1.68 | [M+H]^+^ | 374.0991, 375.1024 | 8-Oxoepiberberine | Organoheterocyclic compounds |
| 87 | C_21_H_21_NO_5_ | 25.11 | 390.1312 | 390.1307 | -1.38 | [M+H]^+^ | 360.0836, 375.1073, 390.1306, 391.1340 | 8-oxoypalMatine | Organoheterocyclic compounds |
| 88 | C_16_H_12_O_5_ | 25.78 | 285.0758 | 285.0753 | -1.54 | [M+H]^+^ | 270.0521, 285.0755, 286.0785 | Wogonin | Flavonoids |
| 89 | C_15_H_10_O_4_ | 26.47 | 299.0561 | 299.0553 | -2.78 | [M-H]^-^ |  | Chrysin | Flavonoids |
| 90 | C_16_H_12_O_5_ | 26.71 | 285.0758 | 285.0755 | -0.88 | [M+H]^+^ | 270.0518, 285.0751, 286.0781 | Oroxylin A | Flavonoids |
| 91 | C_19_H_13_NO_5_ | 26.71 | 358.0686 | 358.0681 | -1.26 | [M]^+^ | 358.0678, 359.0709 | 8-Oxocoptisine | Organoheterocyclic compounds |
| 92 | C_20_H_17_NO_5_ | 27.09 | 374.0999 | 374.0995 | -1.12 | [M+H]^+^ | 344.0521, 359.0756, 374.0990, 375.1020 | Oxyberberine | Organoheterocyclic compounds |
| 93 | C_26_H_30_O_7_ | 28.66 | 455.2064 | 455.206 | -1.03 | [M+H]^+^ | 161.0598, 409.2009, 411.2177, 419.1851, 455.2058 | Obacunone | Terpenes |
| 94 | C_17_H_14_O_5_ | 29.65 | 299.0914 | 299.0911 | -1.1 | [M+H]^+^ | 255.0641, 283.0591, 284.0677, 299.0911, 300.0933 | Moslosooflavone | Flavonoids |
